# Musashi-2 potentiates colorectal cancer immune infiltration by regulating the post-translational modifications of HMGB1 to promote DCs maturation and migration

**DOI:** 10.1186/s12964-024-01495-z

**Published:** 2024-02-12

**Authors:** Xiaole Meng, Risi Na, Xiao Peng, Hui Li, Wanxin Ouyang, Wenting Zhou, Xuting You, Yuhuan Li, Xin Pu, Ke Zhang, Junjie Xia, Jie Wang, Huamei Tang, Guohong Zhuang, Zhihai Peng

**Affiliations:** 1 Organ Transplantation Institute of Xiamen University; Xiamen Human Organ Transplantation Quality Control Center; Xiamen Key Laboratory of Regeneration Medicine; Fujian Provincial Key Laboratory of Organ and Tissue Regeneration, School of Medicine, Xiamen University, Xiamen, Fujian 361102 China; 2 Xiamen Clinical Research Center for Cancer Therapy; Department of Pathology, Zhongshan Hospital (Xiamen Branch), Fudan University; National Institute for Data Science in Health and Medicine, Xiamen University, Xiamen, Fujian 361102 China; 3 Department of Pathology, Xiang’an Hospital of Xiamen University, School of Medicine, Xiamen University, Xiamen, Fujian 361102 China; 4 Organ Transplantation Clinical Medical Center of Xiamen University; Department of General Surgery, Xiang’an Hospital of Xiamen University, School of Medicine, Xiamen University, Xiamen, Fujian 361102 China

**Keywords:** Musashi-2, HMGB1, Post-translational modifications (PTMs), Immune infiltration, Colorectal cancer (CRC)

## Abstract

Post-translational modifications (PTMs) of the non-histone protein high-mobility group protein B1 (HMGB1) are involved in modulating inflammation and immune responses. Recent studies have implicated that the RNA-binding protein (RBP) Musashi-2 (MSI2) regulates multiple critical biological metabolic and immunoregulatory functions. However, the precise role of MSI2 in regulating PTMs and tumor immunity in colorectal cancer (CRC) remains unclear. Here, we present data indicating that MSI2 potentiates CRC immunopathology in colitis-associated colon cancer (CAC) mouse models, cell lines and clinical specimens, specifically via HMGB1-mediated dendritic cell (DC) maturation and migration, further contributes to the infiltration of CD4^+^ and CD8^+^ T cells and inflammatory responses. Under stress conditions, MSI2 can exacerbate the production, nucleocytoplasmic transport and extracellular release of damage-associated molecular patterns (DAMPs)-HMGB1 in CRC cells. Mechanistically, MSI2 mainly enhances the disulfide HMGB1 production and protein translation via direct binding to nucleotides 1403–1409 in the HMGB1 3′ UTR, and interacts with the cytoplasmic acetyltransferase P300 to upregulate its expression, further promoting the acetylation of K29 residue in HMGB1, thus leading to K29-HMGB1 nucleocytoplasmic translocation and extracellular release. Furthermore, blocking HMGB1 activity with glycyrrhizic acid (Gly) attenuates MSI2-mediated immunopathology and immune infiltration in CRC in vitro and in vivo. Collectively, this study suggests that MSI2 may ﻿improve the prognosis of CRC patients by reprogramming the tumor immune microenvironment (TIME) through HMGB1-mediated PTMs, which ﻿might be a novel therapeutic option for CRC immunotherapy.

**Supplementary Information:**

The online version contains supplementary material available at 10.1186/s12964-024-01495-z.

## Introduction

As one of the most common human malignancies, colorectal cancer (CRC) accounts for approximately 10% of the worldwide cancer incidence and mortality [1]. Despite recent advances in delineating the mechanisms involved in colorectal carcinogenesis and cancer therapy, some patients still experience tumor recurrence or metastasis after treatment [2]. Recently, accumulating evidence has demonstrated that the tumor immune microenvironment (TIME) and immune infiltration are crucial to the prognosis and outcome of CRC patients [3, 4], and immunotherapy also holds great promise as a strategy for cancer treatment. However, the efficacy of cancer immunotherapy in patients remains limited [5], and the crosstalk between tumor cells and infiltrating immune cells in the CRC TIME remains incompletely understood. Therefore, addressing these uncertainties to improve immunotherapeutic strategies and the prognosis of CRC is warranted.

RNA-binding proteins (RBPs) are pivotal in the regulation of basic cellular processes, such as RNA splicing, localization, modification, degradation, transport, stability and translation [6]. The RBP Musashi-2 (MSI2), a member of the Musashi gene family, has been implicated in various physiological and pathobiological processes, such as controlling cell differentiation [7, 8], tissue regeneration [9] and cancer development [10]. Recent studies have indicated that MSI2 has gained considerable traction in the context of immune responses and tumor metabolic processes. Focusing not on the main function of MSI2 in RNA processing but on modulation of immune responses, Kim et al. [11] revealed the close association between MSI2 methylation and systemic allergic inflammation, Chen et al. [12] showed that MSI2 knockout decreased myofibroblast-derived IL-6 and IL-11 secretion, and MSI2 was also involved in immune responses and inflammatory infection pathways in HCT116 and A549 cells, such as TNF-α, IL-17, and NF-κB signaling pathways [13]. Regarding tumor metabolism, MSI2 could increase the translation and stability of targeted proteins to alter amino acid metabolism [14] and glycolipid metabolism processes [15]. Moreover, our previous studies have also identified the other unique roles of MSI2 in immunometabolism and post-translational modifications (PTMs); specifically, we observed that MSI2 positively regulated metabolic reprogramming and immune infiltration in renal clear cell carcinoma, which can be used to predict the sensitivity of targeted therapy and immunotherapy [16]. In addition, MSI2 knockout in type 3 (RORgt^+^) innate lymphoid cells in mice attenuated dextran sodium sulfate (DSS)-induced colitis pathophysiology [17], and USP10 could interact with and stabilize MSI2 through Lys48-linked deubiquitination to prevent its degradation in CRC [18], also MSI2 knockdown could trigger CRC cell ferroptosis progression [19]. Despite these findings, the precise immune function and underlying mechanism of MSI2 in the CRC TIME, especially regarding tumor immunity and metabolism, have not been documented. Hence, further investigation is required to elucidate the underlying mechanism of MSI2 in CRC tumor immunometabolism.

PTMs, especially lysine acetylation of histone proteins and non-histone proteins, influence a myriad of key cellular processes related to physiology and disease [20, 21]. High-mobility group protein B1 (HMGB1) is one of the most abundant non-histone nuclear proteins that can serve as an alarmin or damage-associated molecular pattern (DAMP) to drive the pathophysiological processes of inflammatory and immune diseases [22]. Importantly, the biological activity of HMGB1 depends on its cellular localization context, redox state and PTMs [23]. Cell stress and infection have been found to be able to stimulate the translocation of HMGB1 from the nucleus to the cytoplasm and its subsequent release into the extracellular milieu [24]. In addition, HMGB1 can be passively released from dying cells or actively secreted extracellularly in response to danger signals [25], triggering further activation of proinflammatory cascades and recruitment of immune cells. Furthermore, recent consensus holds that the major isoforms of HMGB1 are the reduced, disulfide, thiol and oxidized isoforms; ﻿﻿the reduced, disulfide and thiol HMGB1 isoforms are suggested to be proinflammatory cytokine-like or chemokine-like molecules that recruit inflammatory cells to promote immune activation and clearance, while the oxidized HMGB1 isoform acts as an inducer to promote immune tolerance [26–30]. Moreover, PTMs, such as acetylation, methylation, phosphorylation, ADP-ribosylation, lactylation and S-nitrosylation, have been shown to govern the cellular localization of HMGB1 and its immunometabolic functions involved in inflammatory diseases [31–34]. In addition, acetylation of critical lysine residues located in nuclear localization sequences (NLSs) is the most common PTM for regulating HMGB1 cytoplasmic accumulation [35, 36], and multiple lysine acetyltransferases (KATs) and lysine deacetylases (KDACs) have been confirmed to regulate HMGB1 PTMs [32, 37–39]. Additionally, previous studies have shown that various RBPs can interact with and control the release of HMGB1 to induce inflammatory responses [40]. However, the mechanism by which the RBP MSI2 regulates HMGB1 PTMs is still largely unknown.

In the present study, we investigated that MSI2 can enhance disulfide HMGB1 translation and interact with the cytoplasmic acetyltransferase P300 to upregulate its expression, further promoting the acetylation of K29 residue in HMGB1, thus leading to K29-HMGB1 nucleocytoplasmic translocation and extracellular release and in turn resulting in dendritic cell (DC) maturation and migration, which further contribute to immune infiltration and the inflammatory response, and HMGB1 blockade can attenuate MSI2-mediated immunopathology and immune infiltration. These findings highlight the importance of MSI2 in controlling the PTMs of HMGB1 that mediate immune infiltration in CRC.

## Materials and methods

### Human subjects

This study used a clinical cohort of paired CRC tumor tissues and corresponding normal adjacent tissues from 50 patients that were collected at Xiang’an Hospital of Xiamen University. The Ethics Committee of Xiang’an Hospital of Xiamen University approved this study (Approval number: 20200722YJ001), which was carried out in accordance with the Declaration of Helsinki, and all patients and donors signed informed consent forms.

### Mouse information

Msi2^*Transgenic*^ mice (expressing the *Villin* promoter, pBR322- villin-TRE-Msi2-polyA Vector) were generated by Shanghai Model Organisms Center. Transgenic mice were verified by western blotting, immunofluorescence staining and genotyping. Mouse genotyping was performed by 2% agarose gel electrophoresis of PCR products amplified from DNA isolated from mouse tail biopsies. The sequence of the forward primer used for genotyping was 5′- GCTCGACGGCCACTGCTCTC-3′, and that of the reverse primer was 5′- CTTCTTTCGGCTGAGCTTTCTTAC-3′. This primer pair generated a 606-bp PCR product. WT C57BL/6 mice were used as controls in untreated and CAC experiments. For CAC induction, treatment with the carcinogen AOM (Sigma, Cat. A5486) and repeated DSS (MP Biomedicals, Cat. 160,110, mol wt. 36–50 kDa) treatments were scheduled as follows: mice (8–10 weeks old) were first injected intraperitoneally with a single dose of AOM (10 mg/kg) and were then subjected to 3 cycles of treatment with 3% DSS via feeding. For HMGB1 blockade in vivo, transgenic mice were injected intraperitoneally with Gly (MCE, Cat. HY-N0184) (50 mg/kg) twice during each DSS treatment period in the CAC model. Colon tissues and lymphocytes of the intestinal CLP, spleen and MLNs were then monitored. All mice were maintained and bred at the pathogen-free facility of Xiamen University Laboratory Animal Center. All experimental procedures were performed in accordance with the Guide for the Care and Use of Laboratory Animals and approved by the Xiamen University Laboratory Animal Center (Approval number: XMULAC20190113).

### Cell transfection and generation of stable cell lines

Plasmids were transfected using Lipofectamine 2000 (Invitrogen, Cat. 11,668,019). ShMSI2, OE and control plasmids in recombinant lentiviral vectors were synthesized by GeneChem (Shanghai, China). The shRNA sequences are described below: shMSI2#1 (5′-GTGGAAGATGTAAAGCAATAT-3′) and shMSI2#2 (5′-CCCAACTTCGTGGCGA CCTAT-3′). After a large number of plasmids was purified to be endotoxin-free, lentivirus infection was used to produce lentiviruses expressing the constructs for stable MSI2 knockdown or overexpression in HEK 293 T cells. Viral supernatants were harvested 48 h after transfection and were then used to infect CRC cell lines. Transduced cells were selected with 2 μg/mL puromycin for 15 days, and the transduction efficiency was then determined using qRT–PCR and Western blot analyses.

### Cell culture and reagents

The human CRC cell lines SW620, LOVO, HT29, RKO, SW1116, CACO2, SW480, and HCT116 and HEK 293 T cells were purchased from the Type Culture Collection of the Chinese Academy of Sciences (Shanghai, China) with certification by short tandem repeat (STR) profiling. These cells were cultured in Dulbecco’s modified Eagle’s medium (HyClone, #SH30022.01) supplemented with 10% (v/v) fetal bovine serum (Gemini, Foundation, Cat. 900–108-A11H00K) and 1% penicillin (100 U/mL)/streptomycin (100 ng/mL) (HyClone, #SV30010). For the stress experiment, stable SW620 and LOVO cells were seeded in 6-well plates for 24 h and were then stimulated with 10 μg/mL LPS (Bioss, Cat. bs-8000P) for 0–8 h. For induction of cell death, stable SW620 and LOVO cells were treated with different concentrations of BCL-2 inhibitors (ABT-737 (Beyotime, Cat. SC0015) and ABT-263 (Beyotime, Cat. SC0011)) for 24 h. For precipitation of supernatant proteins, culture medium of stable cells was harvested, incubated with 20% (m/v) cold trichloroacetic acid (TCA) on ice for 1 hour, and then centrifuged at 12,000×g for 10 min at 4 °C, and the pellets were washed twice with cold acetone and dried on ice. For Gly blockade in vitro, stable SW620 and LOVO cells were treated with Gly (MCE, Cat. HY-N0184) (1 μM) for 8–12 h. For the histone deacetylase inhibition assay, SW620, LOVO and HEK 293 T cells were treated with TSA (Solarbio, Cat. IT1250) (1 μM) and/or NAM (Solarbio, Cat. SN8120) (10 mM) for 12 h. For the acetyltransferase P300 inhibition assay, SW620 and LOVO stable cells were treated with 10 μg/mL LPS and C646 (Selleck, Cat. S7152) (10 μM) for 8 h. The protein translational inhibition assay was performed as described previously [41], and stable cell lines were treated with 10 μM CHX (Selleck, Cat. S7418) for 2 h, 4 h, and 8 h. Western blotting and qRT–PCR were then used to determine protein and mRNA levels under different conditions. All cells were maintained at 37 °C in a humidified atmosphere containing 5% CO_2_ and confirmed to be free of Mycoplasma contamination.

### RNA extraction and qRT–PCR

Total RNA from mouse and human CRC tissues or cells was extracted using TRIzol reagent (Thermo Fisher Scientific; Invitrogen, Cat. 15,596,018) according to the manufacturer’s instructions. cDNA was synthesized using HyperScript III RT SuperMix for qPCR with gDNA Remover (EnzyArtisan, Cat. R202). qRT–PCR analysis was performed using Universal SYBR qPCR Mix (EnzyArtisan, Cat. Q204), and the relative RNA expression levels were calculated using the 2^-△△Ct^ method. We utilized the human β-actin (ACTB) or mouse Gapdh gene as an endogenous control, and the obtained data were normalized. All primer sequences used were synthesized by Tsingke Biotechnology Co., Ltd. and are listed in Supplementary Table 1.

### Western blot analysis

Western blot analysis was performed as described previously [42]. In brief, cell or mouse colon mucosal tissue lysates were prepared in ice-cold RIPA buffer (Beyotime, Cat. P0013B) containing protease inhibitor cocktail (TargetMol, Cat. C0001) and a phosphatase inhibitor (1 mM PMSF). The lysates were centrifuged at 12,000×g for 10 min at 4 °C. Cell and supernatant protein concentrations were quantified with a Pierce BCA protein assay kit (Thermo Fisher). For DTT reduction, proteins in stable cell lysates were denatured at 100 °C for 10 min with loading buffer (denaturing, nonreducing, 5×) (Epizyme, Cat. LT103) supplemented with 2 mM DTT (Solarbio, Cat. D8220), as the Oxidized and Disulfide controls, the Human Recombinant HMGB1 (Novoprotein, Cat. C357) was exposed to either 1 and 5 mM DTT or H_2_O_2_ for 30 minutes. Western blotting was carried out using standard techniques with the antibodies listed in Supplementary Table 2.

### Nuclear-cytoplasmic fractionation

Stable SW620 or LOVO cells (1 × 10^7^) were washed twice with cold PBS and precipitated via centrifugation at 4000 rpm for 3 min at 4 °C. Nuclear and cytoplasmic proteins were extracted using NE-PER Nuclear and Cytoplasmic Extraction Reagents (Thermo Scientific, Cat. 78,833) according to the manufacturer’s instructions. The abundances of nuclear and cytoplasmic proteins were then determined by Western blotting, and Laminb or Histone-3 served as the internal reference for nuclear proteins.

### Bio-plex pro mouse cytokine 23-plex immunoassay

For multiplex serum cytokine detection, mouse blood samples were obtained via retro-orbital blood collection and were then centrifuged at 12,000×g for 15 min at 4 °C. The supernatants were collected, and the concentrations of 23 mouse cytokines were determined using a Bio-Plex Pro Mouse Cytokine 23-Plex Immunoassay (Bio-Rad, Hercules, Cat. M60009RDPD/64465244) according to the manufacturer’s specifications.

### ELISA assay

HMGB1 can be actively secreted into the extracellular space or passively released into the external environment; therefore, we employed ELISA to measure the HMGB1 concentration in the supernatants and cytoplasmic lysates of stable CRC cells. The cytoplasmic lysates and supernatants of stable cells were harvested after 8–12 h of stimulation with or without LPS, and a Human High Mobility Group Protein B1 (HMGB-1) ELISA Kit (Cusabio, Cat. CSB-E08223h) was used according to the manufacturer’s instructions.

### Immunoprecipitation (IP) assay

For the IP assay, cell lysate supernatants were immunoprecipitated with 2 μg of the indicated antibodies for 8 h at 4 °C, after which 50 μl of Protein A/G IP/Co-IP magnetic beads (Epizyme, Cat. YJ201) were added and incubation was continued for 4–6 h at 4 °C. The immunocomplex-coupled beads were then washed six times with IP buffer containing PMSF. Finally, the immunocomplex-coupled beads were resuspended in loading buffer for 10 min at 100 °C and analyzed by Western blotting.

### Flow cytometric analysis

Flow cytometric analysis was performed as described previously [43]. In brief, single-cell suspensions were isolated from spleens or MLNs, erythrocytes were removed by hypotonic lysis (Solarbio, #R1010), and dead cells were excluded by Fixable Viability Dye eFluor 780 (eBioscience) staining. For analysis of surface markers, cells were stained in PBS containing 2% bovine serum albumin (BSA) and 2 mmol/L EDTA. Single lymphocytes from the intestinal mucosa lamina propria were obtained by digestion with collagenase VIII (Sigma–Aldrich, Cat. C5138) and deoxyribonuclease I (Sigma–Aldrich, Cat. DN25) for 60 min, and the supernatants were then passed through a 70 μm cell strainer and subjected to Percoll (GE Healthcare) density gradient centrifugation. The FACS antibodies used are listed in Supplementary Table 3. The stained cells were then analyzed with a Beckman CytoFlex flow cytometer (Beckman, USA).

## Human PBMC isolation, iDC induction and coculture

PBMCs were isolated from healthy donor whole blood by a density gradient centrifugation method using Ficoll (GE Healthcare). iDCs were generated by adding recombinant human GM-CSF (80 ng/mL) (Novoprotein, Cat. C003) and recombinant human IL-4 (40 ng/mL) (Novoprotein, Cat. CX03) to the isolated PBMCs and culturing for 9 days according to the previous study [44]. For the coculture assay, iDCs and stable cells at a ratio of 5:1 (5 × 10^5^ cells:1 × 10^5^ cells) were mixed in culture with or without rh-HMGB1 (50 ng/mL) (Novoprotein, Cat. C357) for 24 h. iDCs were then collected for flow cytometry to evaluate DC maturity.

### Immunofluorescence (IFC) analysis

IFC analyses were performed as described previously [45]. In brief, for analysis of tissues, CRC sections were deparaffinized, subjected to antigen retrieval in Tris-EDTA buffer (pH 9.0) at 95 °C for 40 min, and then blocked with 10% normal goat serum at room temperature for 60 min. For analysis of stable cells, cells were seeded on coverslips in 24-well plates and fixed with 4% paraformaldehyde for 15 min after two washes with PBS at room temperature. Then, the cells were permeabilized in 0.5% (v/v) Triton X-100 for 10 min after five washes with PBS. The coverslips were then incubated with blocking buffer containing 5% BSA. Afterward, the sections and cells were incubated with primary antibodies at 4 °C for 8 h. After five washes with PBST (0.1% Tween 20 in PBS), the sections and cells were incubated with Alexa Fluor®-conjugated secondary antibodies (Abcam), and nuclei were stained with DAPI. The coverslips and sections were washed five times, and images were acquired using a Nikon A1R confocal microscope.

### Dual-luciferase reporter assay

To construct wild-type plasmids, cDNA corresponding to a 200 bp fragment of the HMGB1 3’UTR was inserted into the pmirGLO vector (Promega). To construct mutant plasmids, the TAGTTAG sequence in the 3’UTR of HMGB1 mRNA was replaced with AAAAAAA. The sequences of the wild-type and mutant 200-bp HMGB1 3’UTR sequences can be found in Supplementary Table 4. Then, the wild-type or mutant HMGB1 plasmid was cotransfected with the MSI2-Flag plasmid into HEK 293 T cells. After 48 h, cells were harvested to evaluate luciferase activity according to the manufacturer’s protocol.

### 4D label-free proteomics, acetylomics and quantitative analysis

The 4D label-free proteomics and acetylomics analyses were carried out by PTM Biolab (PTM Bio; Hangzhou, China). For acetylomics, anti-acetyllysine antibody-conjugated agarose beads (PTM-104, PTM Bio) were incubated with prewashed tryptic peptides. The differentially expressed proteins identified by proteomics analysis of stable SW620 and LOVO cells are reported in Supplementary Datasheet 1. KEGG pathway enrichment analysis was performed with DAVID Bioinformatics Resources 6.8 (https://david.ncifcrf.gov). The differentially modified sites in the proteins identified by acetylomics analysis of stable SW620 cells are reported in Supplementary Datasheet 2. Genes encoding proteins with differential modification sites were subjected to GO or KEGG enrichment analysis. The GO enrichment annotations were based on biological processes, cellular components and molecular functions. KEGG pathway enrichment analysis using the online service tool KAAS was used to annotate pathways with the descriptions in the KEGG database. For domain annotation analysis, the functional descriptions of the identified protein domains were determined with InterProScan based on protein sequence alignment and the InterPro domain database (http://www.ebi.ac.uk/interpro/). Two-tailed Fisher’s exact test was employed to evaluate the enrichment of the differentially expressed proteins with respect to all identified proteins, and a corrected *p* value of < 0.05 was considered to indicate significance.

### Shotgun LC–MS/MS and differential protein analysis

Shotgun LC–MS/MS was carried out by Shanghai Applied Protein Technology. In brief, tryptic peptides from HT29-OE (with MSI2 overexpression) and HT29-NC cell samples were ionized by a Nano spray ionization (NSI) source prior to MS/MS in Q Exactive TM Plus mass spectrometer (Thermo) coupled online to the ultra-performance liquid chromatography (UPLC) system, and peptides with matching spectra from all datasets were then assembled into proteins. Reactome pathway enrichment analysis was performed with DAVID Bioinformatics Resources 6.8 (https://david.ncifcrf.gov). The differentially expressed proteins are listed in Supplementary Datasheet 3.

### RNA immunoprecipitation (RIP)

SW620 and LOVO cells overexpressing Flag-MSI2 were used for RIP with a Magna RIP RNA Binding Protein Immunoprecipitation Kit (Millipore, Billerica, MA). An anti-MSI2 antibody was used for protein IP, and the RIP assay was carried out according to the manufacturer’s instructions. Then, RNA was extracted using the phenol/chloroform method, and RNA binding to the MSI2 protein was assessed by qRT–PCR. The PCR products were analyzed by 2% agarose gel electrophoresis, and the specific primer sequences for amplification of HMGB1 can be found in Supplementary Table 4.

### RNA pulldown assay

The RNA pulldown assay was performed as described previously [41]. A T7 High Yield RNA Synthesis Kit (Yeasen, Cat. 10623ES50) were used to synthesize the HMGB1 3’UTR RNA (Forward, 5′-CTAATACGACTCACTATAGGTTGA-3′ and Reverse, 5′-TTAACTGATCAAGATTTTGGATGTTCAG-3′). Synthesized RNAs were labeled with biotin and magnetic beads and were then incubated with LOVO cell lysates following the manufacturer’s instructions for the Pierce Magnetic RNA-Protein Pull-Down Kit (Invitrogen, Cat. 20,164). The input, flowthrough and eluted proteins were detected by Western blotting.

### Histopathology

In brief, fresh tissues were fixed with 4% paraformaldehyde at 4 °C overnight and were then paraffin embedded and sectioned. For H&E staining, sections were stained with hematoxylin and dehydrated in an alcohol gradient and xylene. IHC staining of proteins, including MSI2 (Abcam, #ab76148), CD3 (Servicebio, #GB11014), CD11c (Servicebio, #GB11059), CD11b (Servicebio, # GB11058), CD68 (Servicebio, # GB113150), Ly6g (Servicebio, # GB11229), P300 (Affinity, #AF5360) and HMGB1 (Abcam, #ab228624), was performed as described previously [16], and staining was analyzed by calculating the mean density per area using Image-Pro Plus. Histological analysis was performed to determine the inflammation score, which was defined as follows (modified from a previous report) [46]. The score for the depth of inflammatory infiltration was defined as follows: no inflammatory infiltrate in the lamina propria, 0; increased presence of inflammatory cells in the mucosa, 1; inflammatory infiltrate extending into the submucosa or existing necrotic cell death in the crypt lumen, 2; and evident transmural extension of the inflammatory infiltrate or ulceration, 3. The score for the area of inflammation was defined as follows. A score of 0 indicated the absence of inflammation, and scores of 1, 2, 3, 4, and 5 indicated that the area of inflammation covered less than 10%, 10–20%, 20–50%, 50–70%, and more than 70% of the total tissue section area, respectively. The final inflammation score was calculated by multiplying the inflammation area score by the infiltration depth score. Pathologists blinded to the specific experimental conditions determined each score from 10 random visual fields at 20× magnification.

### Bioinformatic and database analysis

The TCGA CRC database was analyzed with GEPIA (http://gepia2.cancer-pku.cn/) to determine the correlations between the expression of MSI2 and that of inflammation-associated genes and DC maturation markers by using *Spearman* correlation analysis and normalized by GAPDH. Online-available GSE14333 and GSE164191 datasets were downloaded from GEO (https://www.ncbi.nlm.nih.gov/geo/) to determine the correlations between the expression of MSI2 and that of inflammatory genes by using *Spearman* correlation analysis. TCGA datasets were downloaded from TCGA (https://portal.gdc.cancer.gov/) to analyze the differentially expressed genes in MSI2-high and MSI2-low patients, and the differentially expressed genes were then further subjected to GO and KEGG pathway enrichment analyses. “Adjusted p < 0.05 and Log2(Fold Change) >1.5 or < −1.5” were defined as the threshold criteria for differential mRNA expression. *Spearman* correlation coefficients were used to determine the correlations between the expression of MSI2 and that of KAT and KDAC genes. The distribution of the immune score by MSI2 expression was used in immuneeconv/TIMER to evaluate tumor immune infiltration in MSI2-high and MSI2-low patients. Overall survival curves were plotted using the Kaplan–Meier method, and survival differences were analyzed with the log-rank test; univariate and multivariate Cox regression analyses were used to estimate 5-year overall survival; and a Nomogram model was used to predict the 1-year, 2-year, 3-year and 5-year overall survival of CRC patients based on MSI2 expression. The CPTAC CRC (https://proteomics.cancer.gov/programs/cptac) database was employed to analyze the protein expression of MSI2 and HMGB1 in primary CRC and normal tissues. TIMER (https://cistrome.shinyapps.io/timer/) was employed to analyze the associations between MSI2 expression and immune cell infiltration, and the purity-corrected partial *Spearman* correlation coefficients are shown in scatterplots. All analysis methods and R packages were implemented in R version 4.0.3 (R Foundation for Statistical Computing, Vienna, Austria), and the ggstatsplot, ggplot2 and pheatmap packages in R were used to draw each figure.

### Statistical analysis

Data analyses were conducted using GraphPad Prism 7.5, FlowJo v10, ZEN 2.3 Lite and Image-Pro Plus software. Two-tailed unpaired or paired Student’s t test and the Mann–Whitney test were used for comparisons between two groups. One-way ANOVA was used for comparisons among more than two groups. All quantitative data are presented as the mean ± standard deviation of three independent experiments. A *p* value of < 0.05 was considered to indicate a statistically significant difference. *p < 0.05, **p < 0.01, ***p < 0.001, ****p < 0.0001; ns: not significant.

## Results

### MSI2 plays a critical role in CRC immunopathology

**Fig. 1:**
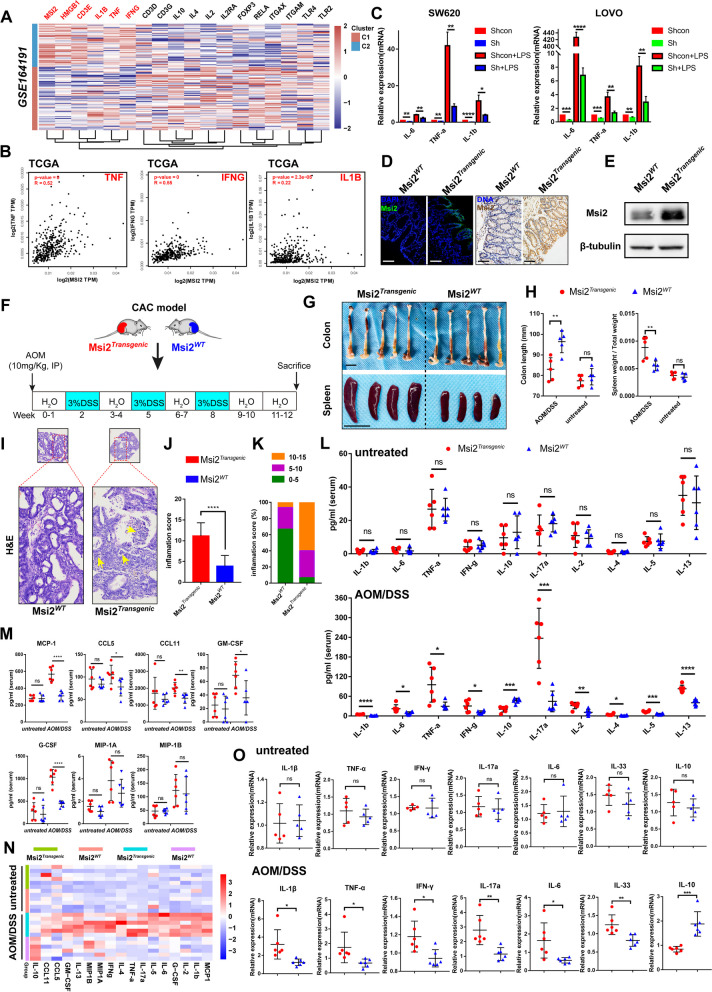
MSI2 plays a critical role in CRC immunopathology. **A** Heatmap showing the expression of MSI2 and inflammatory factors in the GSE164191 CRC dataset. **B** In GEPIA CRC database, S*pearman* correlation analysis was used to identify positive correlations between MSI2 expression and TNF (*R* = 0.52), IFNG (*R* = 0.55), and IL1B (*R* = 0.22) expression. **C** The mRNA expression of IL-6, TNF-α, and IL-1β was measured by qRT–PCR in stable SW620 and LOVO cells in the absence or presence of LPS (10 μg/mL) for 8 h. **D** Representative IFC and IHC images of Msi2 expression in Msi2^*Transgenic*^ and WT mice. Scale bars, 100 μm. **E** Western blot analysis of Msi2 expression in Msi2^*Transgenic*^ and WT mice. **F** Schematic diagram of the AOM/DSS treatment protocol to induce colitis-associated cancer (CAC). **G** Representative images of colon and spleen in AOM/DSS induced CAC mice models. Colon (top) and spleen (bottom). Scale bars, 10 mm. **H** Statistical analysis of colon length and spleen weight in CAC and untreated mice. *n* = 5. **I** Representative images of H&E staining of colons from mice with AOM/DSS-induced CAC. The yellow arrows indicate necrotic debris and infiltrated immune cells. Scale bars, 50 μm and 100 μm. **J-K** Histological inflammation score analysis for CAC mice, *n* = 5. **L-M** Statistical analysis of serum cytokines and chemokines in untreated and CAC mice, *n* = 6. **N** Heatmap of different cytokines and chemokines levels in serum using Bio-Plex Pro Mouse Cytokine 23-Plex immunoassay from untreated and CAC mice. **O** The mRNA levels of colonic inflammation-associated genes were measured by qRT–PCR in untreated (*n* = 5) and CAC mice (*n* = 6). These results are presented as the mean ± SD values; ns: not significant, *p < 0.05, **p < 0.01, ***p < 0.001, ****p < 0.0001; **C**,** H**,** M-O** unpaired 2-tailed Student’s t test and **J** Mann–Whitney test

Our previous studies identified that MSI2 is involved in metabolic reprogramming-mediated immune infiltration in ccRCC [16] and immune dysregulation in DSS-induced colitis [17]. And recent study also confirmed that MSI2 can regulate immune-related signaling pathways in HCT116 and A549 cells, including TNF-α, inflammatory response and IFNG response pathway [13]. To determine the immune-related effects of MSI2 in CRC, we first identified the positive correlations between the expression of MSI2 and immune-related factors in the GSE164191 and GSE14333 CRC datasets, such as TNF, IFNG and IL1B, and further confirmed the results by association analysis in TCGA data from GEPIA (Fig. 1A-B and Fig. S1A). We then examined MSI2 expression in CRC and constructed stably transfected cell lines (Fig. S1B-D), and found that MSI2 promoted the production of inflammatory cytokines, including TNF-α, IL-1β and IL-6, in both the presence and absence of LPS (Fig. 1C). Next, to investigate the immune functions of MSI2 in CRC in vivo, transgenic mice with overexpression of Msi2 driven by the *Villin* promoter were generated and validated (Fig. 1D-E and Fig. S2A-B). There were no significant differences in colon length and spleen weight between the groups of untreated mice (Fig. 1H and Fig. S2C). Nevertheless, histologically, only the colons from Msi2^*Transgenic*^ mice showed mild focal inflammation in untreated mice (Fig. S2D). Consistent with the above finding, there were no significant changes in serum and colonic inflammatory cytokines and chemotactic factors in untreated Msi2^*Transgenic*^ mice compared to untreated wild-type (WT) mice (Fig. 1L-O). However, in the AOM/DSS induced CAC model (Fig. 1F), Msi2^*Transgenic*^ mice had significantly reduced colon lengths and enlarged spleens (Fig. 1G-H). Moreover, histological analysis revealed inflammatory necrosis in the colonic crypt lumen, with a large number of infiltrating immune cells in the tumor stroma and mucosal lamina propria (Fig. 1I). Evaluation of histological inflammation scores also revealed more severe immunopathology in Msi2^*Transgenic*^ mice than in WT mice following AOM/DSS treatment (Fig. 1J-K and Fig. S2D). In addition, the Bio-Plex Pro multiplex cytokine assay demonstrated that the serum levels of IL-1β, TNF-α, IL-6, IFN-γ, IL-17a and other inflammatory cytokines were significantly higher in Msi2^*Transgenic*^ CAC mice than in WT CAC mice; similar results were observed for chemokines such as MCP-1, CCL5, CCL11 and G-CSF, but the level of IL-10 was reduced (Fig. 1L-N). Consistent with the above findings, increased colonic mRNA levels of inflammatory cytokines such as IL-1β, TNF-α, IL-6, IFN-γ, IL-33, and IL-17a and decreased IL-10 were observed in Msi2^*Transgenic*^ CAC mice, but no differences in untreated mice (Fig. 1O). Collectively, these findings suggest that MSI2 may play a pivotal role in CRC immunopathology.

### MSI2 promotes CRC immune infiltration by upregulating HMGB1-mediated DC maturation and migration

**Fig. 2:**
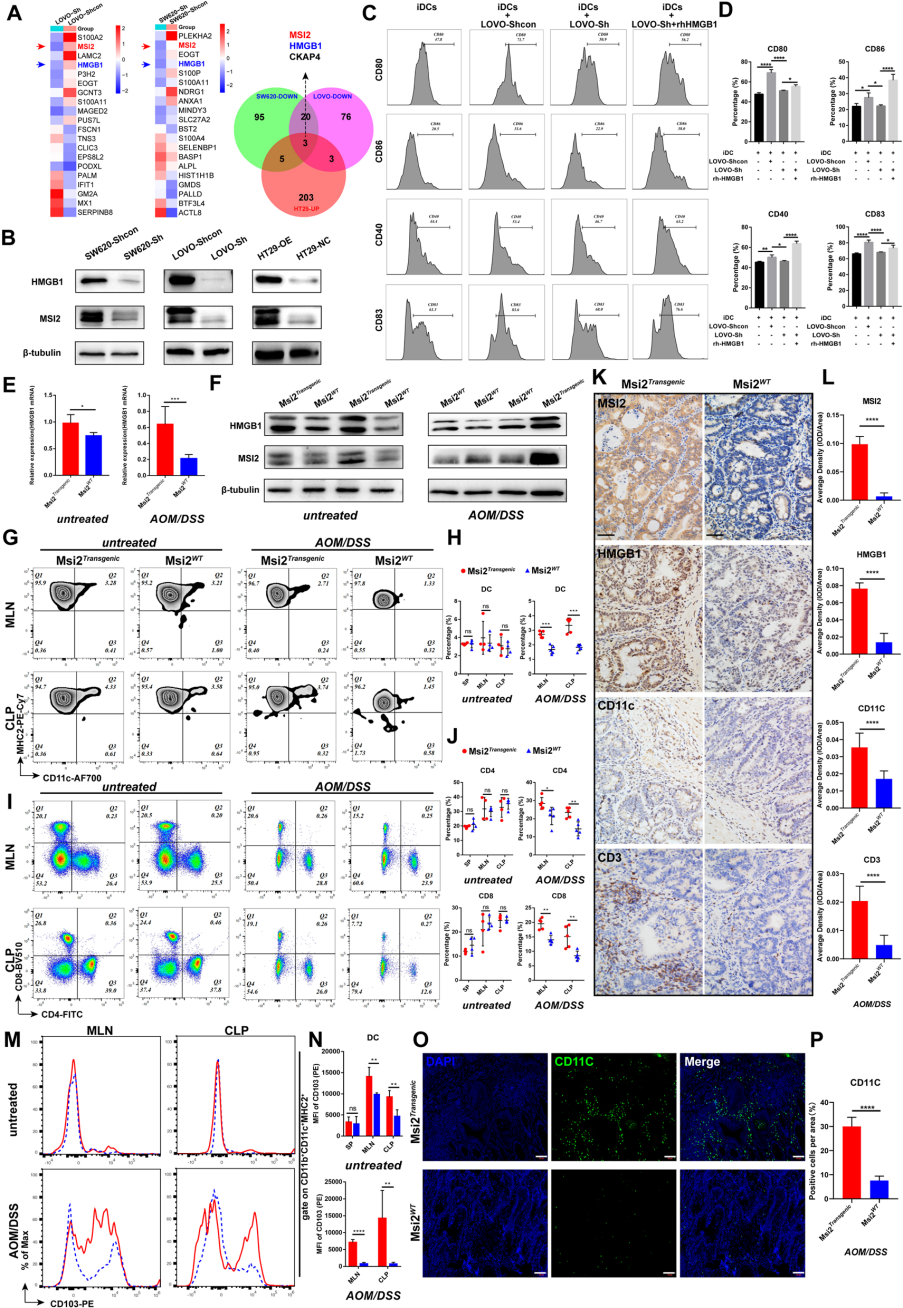
MSI2 modulates tumor immunity in CRC via HMGB1-mediated DC maturation and migration. **A** Enrichment analysis of the differentially expressed proteins showed that MSI2 regulated HMGB1 expression in stable LOVO, SW620, and HT29 cells. Red arrows, MSI2; blue arrows, HMGB1. **B** Western blot analysis of MSI2 and HMGB1 expression in stable LOVO, SW620, and HT29 cells. **C** FACS analysis of the DC maturation markers CD80, CD86, CD40, and CD83 after coculture of iDCs with stable LOVO cells and rh-HMGB1 for 24 h. **D** Statistical analysis of the expression of the DC maturation markers CD80, CD86, CD40, and CD83. **E** HMGB1 mRNA expression was determined by qRT–PCR in untreated and CAC mice, *n* = 5. **F** Western blot analysis of MSI2 and HMGB1 expression in colonic mucosa from untreated and CAC mice. **G-H** FACS and statistical analysis of DCs (CD11b^+^CD11c^+^MHCII^+^) isolated from the SP, MLNs, and CLP of untreated (*n* = 4) and CAC (*n* = 5) mice. **I-J** FACS and statistical analysis of CD4^+^ T and CD8^+^ T-cell percentages among cells isolated from the SP, MLNs, and CLP of untreated (*n* = 4) and CAC (*n* = 5) mice. **K** Representative IHC images of MSI2, HMGB1, CD11c and CD3 staining in CAC mice. **L** Statistical analysis for MSI2, HMGB1, CD3 and CD11c average IHC staining density (*n* = 5). Scale bars, 100 μm. **M** FACS analysis of CD103 expression with gating on DCs (CD11b^+^CD11c^+^MHCII^+^) in cells isolated from MLNs and CLP of untreated and CAC mice. **N** Statistical analysis of the CD103 mean fluorescence intensity (MFI) in DCs isolated from the SP, MLNs, and CLP of untreated (*n* = 4) and CAC (*n* = 5) mice. **O-P** Representative IFC images and statistical analysis of CD11c-positive cells in Msi2^*Transgenic*^ and WT CAC mice. Scale bars, 100 μm. These results are presented as the mean ± SD values; ns: not significant, *p < 0.05, **p < 0.01, ***p < 0.001, ****p < 0.0001; **D** one-way ANOVA, **L** Mann-Whitney test and **E, H, J, N, P** unpaired 2-tailed Student’s t test

To clarify the mechanism by which MSI2 modulates CRC immunopathology, we first investigated differentially expressed proteins in stable LOVO and SW620 cell lines by proteomics analysis and found downregulation of 102 and 123 proteins after MSI2 knockdown, respectively. Among these, the protein levels of both HMGB1 and MSI2 were decreased in Sh groups (Fig. S3A-B). Moreover, the shotgun LC–MS/MS on HT29 cells analysis also revealed abnormal protein accumulation upon overexpression of MSI2 (Fig. S3C-D). Combined differential protein enrichment analysis indicated that HMGB1 expression was MSI2 dependent, and the Western blot and qRT–PCR results further verified this finding (Fig. 2A-B and Fig. S3E-F). HMGB1 is required for DC function and chemotaxis and acts as a key DAMP released into the extracellular milieu, contributing to DC maturation and migration, which triggers inflammatory responses [47, 48]. Thus, further correlation analyses were performed and confirmed that the expression of MSI2 and/or HMGB1 was positively associated with that of DC maturation markers such as CD80, CD86, CD40 and CD83 in GEPIA CRC database (Fig. S4A-D). Next, we sought to determine whether MSI2-mediated HMGB1 alterations have a role in the activation of DCs. We treated isolated healthy donor PBMCs with rh-IL-4 and rh-GM-CSF for 9 days to induce DC differentiation [44] and validate CD11c expression (Fig. S4E-G), and we then cocultured the induced DCs (iDCs) with stably transfected LOVO cells for 24 h. The expression of iDC maturation markers (including CD80, CD86, CD40 and CD83) was detected by FACS, and HMGB1 was found to be required for the promoting effect of MSI2 on DC maturation (Fig. 2C-D). In vivo, we first examined the mRNA and protein levels of HMGB1 in untreated and CAC mice. HMGB1 expression was found to be significantly upregulated in Msi2^*Transgenic*^ mice with or without AOM/DSS treatment (Fig. 2E-F and Fig. S5A). Moreover, FACS analysis of immune infiltration in the spleen (SP), mesenteric lymph nodes (MLNs) and colonic mucosal lamina propria (CLP) isolated from untreated and CAC WT and Msi2^*Transgenic*^ mice revealed that the percentages of the DC (CD11b^+^CD11c^+^MHCII^+^), CD4^+^ T-cell and CD8^+^ T-cell populations were increased in Msi2^*Transgenic*^ CAC mice compared with WT CAC mice but did not differ between untreated WT and Msi2^*Transgenic*^ mice (Fig. 2G-J and Fig. S5B). Immunohistochemistry also confirmed that MSI2 promoted HMGB1 expression and further increase DC and CD3^+^ T-cell infiltration in CAC mice (Fig. 2K-L and Fig. S5A). In addition, we analyzed the migration signatures of CD4^+^, CD8^+^, and DC cells. The proportion of CD103^+^ DCs was increased in both untreated and CAC Msi2^*Transgenic*^ mice compared with the corresponding WT mice (Fig. 2M-N). Similarly, immunofluorescence results also confirmed that the proportion of CD11c-positive cells in Msi2^*Transgenic*^ CAC mice was more infiltrated than in the WT group (Fig. 2O-P). And the proportions of CD103^+^CD4^+^ and CD103^+^CD8^+^ T cells were also increased in Msi2^*Transgenic*^ CAC mice (Fig. S5C-D). Furthermore, the absolute numbers of leukocytes, lymphocytes, monocytes, and neutrophils from the peripheral blood of the eyeball were all increased in Msi2^*Transgenic*^ CAC mice, and the proportion of lymphocytes was also significantly increased in Msi2^*Transgenic*^ CAC mice (Fig. S5E). These data suggest that MSI2 may contribute to immune infiltration by promoting HMGB1-mediated maturation and migration of DCs in CRC.

### MSI2 enforces HMGB1 production, nucleocytoplasmic translocation and extracellular release

**Fig. 3:**
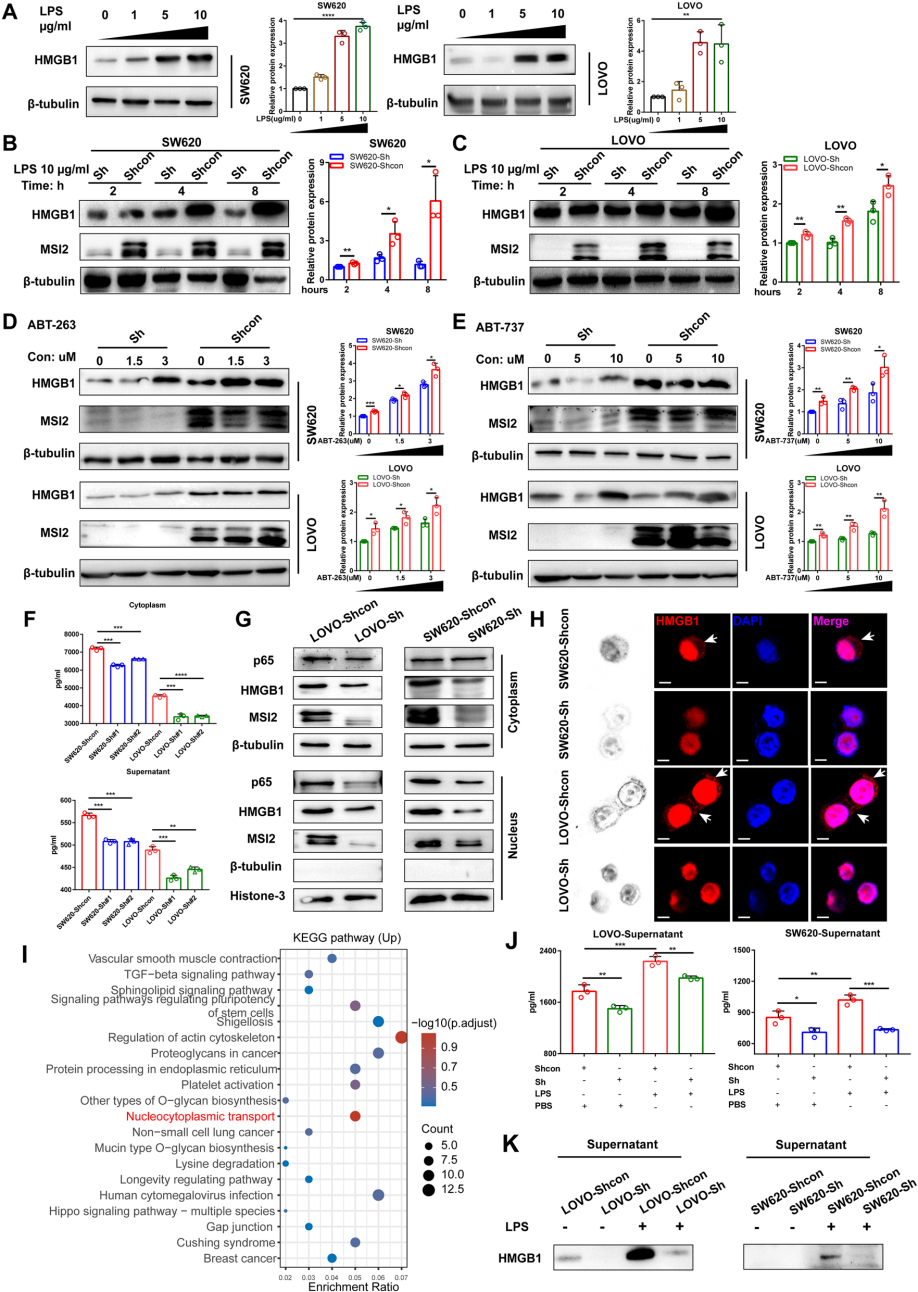
MSI2 enhances HMGB1 production, nucleocytoplasmic translocation and extracellular release. **A** The protein expression of HMGB1 in SW620 and LOVO cells following 8 h of treatment with various concentrations of LPS. **B-C** HMGB1 protein expression in stable SW620 and LOVO cells cultured in the presence of LPS (10 μg/mL) for 2–8 h. **D-E** HMGB1 protein expression in stable SW620 and LOVO cells treated for 12 h with different concentrations of ABT-263 and ABT-737. **F** The concentrations of HMGB1 in the cytoplasm and supernatant of stable SW620 and LOVO cells were determined by ELISA. **G** Western blot analysis showed that loss of MSI2 impeded the translocation of ectopic HMGB1 into the cytoplasm and p65 into the nucleus in stable SW620 and LOVO cells. **H** Representative IFC images of stable SW620 and LOVO cells showing cytoplasmic translocation of ectopic HMGB1, the white arrows indicate cytoplasmic accumulation. Scale bars, 20 μm. **I** The nucleocytoplasmic transport pathway was significantly enriched among the upregulated KEGG pathways in MSI2-high patients in the TCGA CRC database. **J** The concentration of HMGB1 in the supernatant of stable SW620 and LOVO cells treated with LPS (10 μg/mL) or PBS for 8 h was determined by ELISA. **K** The release of HMGB1 into the extracellular supernatant of stable SW620 and LOVO cells treated with or without LPS (10 μg/mL) for 8 h was evaluated. These results are presented as the mean ± SD values; ns: not significant, *p < 0.05, **p < 0.01, ***p < 0.001, ****p < 0.0001; **A-F** unpaired 2-tailed Student’s t test and **J** one-way ANOVA

HMGB1 is a nuclear DNA-binding protein that promotes the inflammatory response when released extracellularly after cellular stress or death [24, 25]. To explore how MSI2 affects HMGB1, we first examined HMGB1 expression in vitro by LPS stimulation or cell death induction. Consistent with previous study [49], HMGB1 expression was upregulated in LPS-stimulated CRC cell lines (Fig. 3A). Then, we evaluated HMGB1 expression in LPS-treated stable LOVO and SW620 cell lines, and Western blot analysis of HMGB1 demonstrated that MSI2 knockdown reduced the protein level of HMGB1 after LPS stimulation (Fig. 3B-C). In addition to passive secretion under stress conditions like LPS exposure, active release of HMGB1 also occurs during cell death. We applied BCL-2 inhibitors to induce cell death and found that HMGB1 expression levels were significantly increased in MSI2 Shcon group cells compared with the corresponding knockdown groups after treatment with ABT-263 or ABT-737 (Fig. 3D-E). Moreover, the cytoplasmic and extracellular concentrations of HMGB1 were also significantly decreased in untreated MSI2 knockdown cells, as measured by ELISA assay (Fig. 3F). Importantly, we found that MSI2 induced nucleocytoplasmic translocation of HMGB1, resulting in the release of HMGB1 in the cytoplasmic milieu, accompanied by intranuclear translocation of p65 (Fig. 3G). These findings were further confirmed by immunofluorescence confocal microscopy (Fig. 3H). In addition, gene set enrichment analysis of RNA-seq data for the MSI2-high and MSI2-low groups of patients from the TCGA CRC datasets (Fig. S6A-B) and KEGG enrichment analysis of the differentially expressed genes indicated that the top-ranked upregulated pathways affected by MSI2 included the nucleocytoplasmic transport signaling pathway (Fig. 3I) and that the upregulated GO terms included regulation of intracellular protein transport, nuclear transport and nuclear export (Fig. S6C). Furthermore, extracellular HMGB1 cytokine concentrations were measured in stable cell lines with or without LPS treatment, and the results of ELISAs and extracellular protein enrichment assays demonstrated that MSI2 promoted HMGB1 release into the extracellular milieu (Fig. 3J-K). Together, these data suggest that MSI2 affects HMGB1 production, nucleocytoplasmic translocation and extracellular release under both unstressed and stress conditions.

### MSI2 interacts with P300 to promote K29-HMGB1 acetylation, translocation and extracellular release

**Fig. 4:**
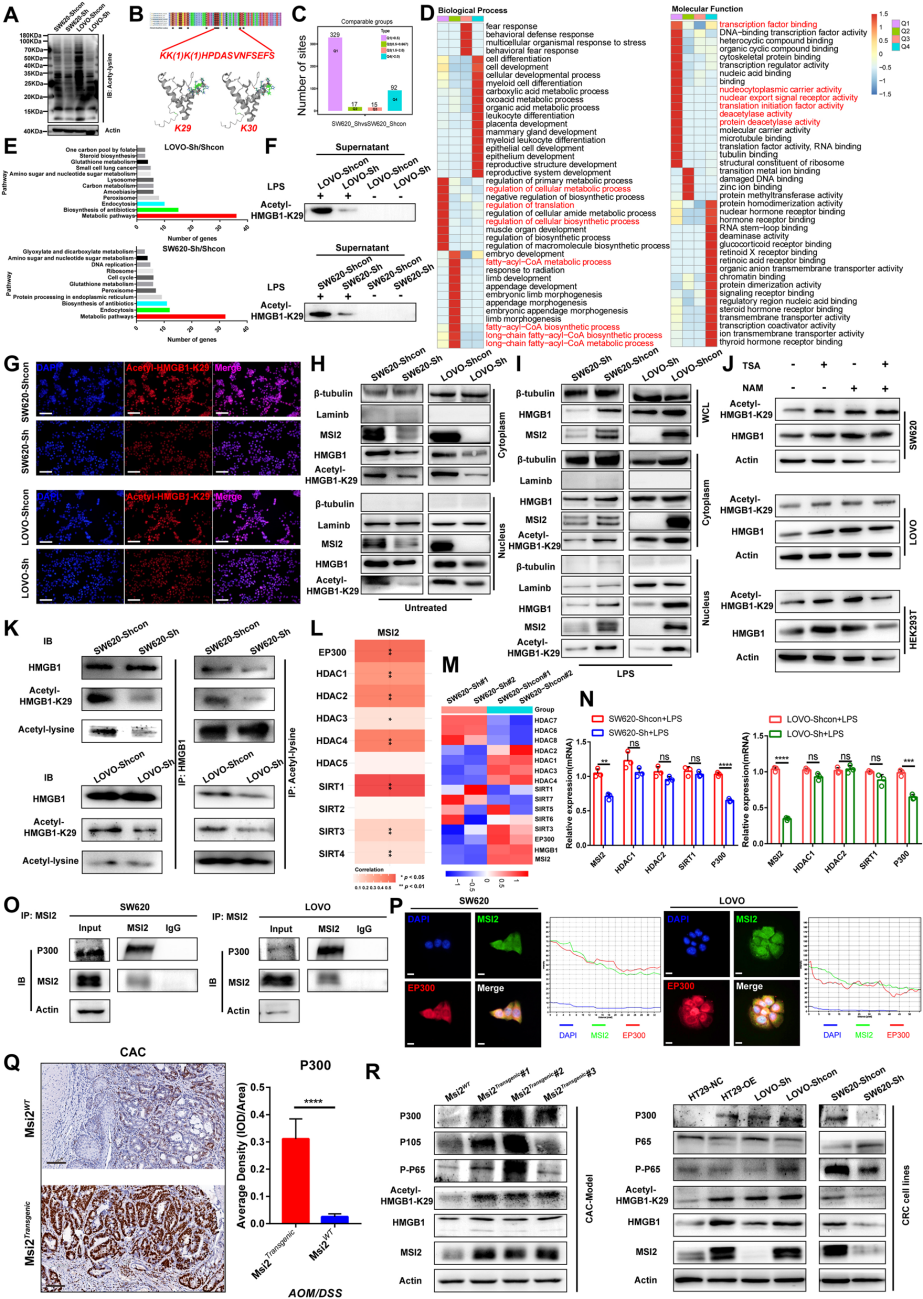
MSI2 interacts with P300 to promote K29-HMGB1 acetylation, translocation and extracellular release. **A** Western blot analysis of acetyllysine in stable SW620 and LOVO cells. **B** Identification of lysine acetylation at K29 and K30 of HMGB1 by 4D label-free acetylome sequencing in stable SW620 cells. **C** Four gene clusters (Q1-Q4) based on the fold difference were identified in stable SW620 cells. **D** KEGG pathway enrichment analysis of the gene clusters (Q1-Q4) by BP and MF. **E** The DEGs identified by proteomics in stable SW620 and LOVO cells were subjected to KEGG pathway enrichment analysis using the DAVID bioinformatics tool. **F** Acetyl-K29-HMGB1 expression in the extracellular supernatant of stable SW620 and LOVO cells treated with or without 10 μg/mL LPS for 8 h. **G** Representative IFC images showing aberrant low expression of Acetyl-K29-HMGB1 in SW620 and LOVO cells with stable MSI2 knockdown. Scale bars, 100 μm. **H-I** Ectopic nucleocytoplasmic translocation of Acetyl-K29-HMGB1 in stable SW620 and LOVO cells in the absence (**H**) or presence (**I**) of LPS (10 μg/mL) for 8 h. **J** Acetyl-K29-HMGB1 and HMGB1 protein levels in HEK 293 T, SW620 and LOVO cells treated with TSA (1 μM) or (and) NAM (10 mM) for 12 h. **K** IP of HMGB1 and acetyllysine in stable SW620 and LOVO cells. **L** Positive correlations between MSI2 expression and KATs and KDACs expression in TCGA CRC database. **M** Heatmap of MSI2 and KAT/KDACs protein expression in stable SW620 cells as determined by 4D label-free proteomics. **N** MSI2, HDAC1, HDAC2, SIRT1 and EP300 mRNA expression was measured by qRT–PCR in stable SW620 and LOVO cells cultured in the presence of LPS (10 μg/mL) for 8 h. **O** Endogenous Co-immunoprecipitation of MSI2 and EP300 in SW620 and LOVO cells. **P** Representative IFC images of MSI2 and EP300 cytoplasmic localization in SW620 and LOVO cells. Scale bars, 10 μm. **Q **Representative IHC images of P300 from CAC mice and statistical analysis for P300 average IHC staining density. Scale bars, 100 μm. **R **Western blot analysis of EP300, HMGB1, K29-HMGB1 and NF-κB gene expression in CAC mice and stable CRC cells. These results are presented as the mean ± SD values; ns: not significant, **p < 0.01, ***p < 0.001, ****p < 0.0001; **N** unpaired 2-tailed Student’s t test, **Q** Mann-Whitney test and **L** *Pearson* correlation analysis

Recent studies have suggested that PTMs of HMGB1, such as acetylation, phosphorylation, ubiquitination, ADP-ribosylation, lactylation and S-nitrosylation, are involved mainly in its translocation and secretion, including its nucleocytoplasmic transport and extracellular release [31–34]. Multiple acetylation-modified residues of HMGB1 have also been confirmed in the UniProt database [36, 50] (Fig. S7A). To elucidate whether the translocation of HMGB1 is responsible for its MSI2-mediated PTM, immunoblot analysis of acetyllysine in stable SW620 and LOVO cells suggested that modulation of MSI2 expression might dysregulate the acetylation process (Fig. 4A). We further performed 4D label-free acetylome sequencing analysis in stable SW620 cells (Fig. S7B). The acetylomics results revealed that 165 and 150 of the differentially modified proteins were localized in the nucleus and cytoplasm, respectively (Fig. S7C). Clusters of COG/KOG category enrichment analysis mainly focuses on translation and PTMs (Fig. S7D). Importantly, we identified the key lysine acetylation sites at K29 and K30 of HMGB1(*KK (1)K(1) HPDASVNFSEFS*) (Fig. 4B), which have been implicated in immune-related disorders [51]. We divided the genes encoding the modification-containing proteins into four gene clusters (Q1-Q4) based on the fold difference and further performed KEGG enrichment analysis, finding that in the biological process (BP) category, the Q1 cluster was enriched mainly in cellular metabolic processes and the regulation of translation processes, the Q2 cluster was enriched mainly in fatty acyl-CoA metabolic and biosynthetic processes and long-chain fatty acyl-CoA metabolic and biosynthetic processes, and the Q4 cluster was enriched mainly in leukocyte differentiation and myeloid cell differentiation. In the molecular function (MF) category, the Q1 cluster was enriched mainly in nucleocytoplasmic carrier activity, transcription factor binding, protein deacetylase activity and nuclear export signal receptor activity (Fig. 4C-D). Moreover, in the Q1 cluster, the enriched cellular component (CC) annotations included mitochondria, nucleolus and cell body; the enriched KEGG pathways included nonalcoholic fatty liver disease, nucleocytoplasmic transport and MAPK signaling pathway; and the enriched protein domains included Myb-like DNA binding domain and C2H2-type zinc finger (Fig. S7E-G). In addition, proteomic differential genes pathway enrichment analysis of stable SW620 and LOVO cell lines revealed aberrant metabolic pathway processes, biosynthesis of antibiotics and endocytosis processes (Fig. 4E). These results provide further evidence that MSI2 can reprogram tumor metabolism, protein translation and immune infiltration [13, 16].

To determine whether the key acetylation site of K29 in HMGB1 is affected by MSI2, we first verified that the release of extracellular acetyl-K29-HMGB1 was increased in the SW620-Shcon and LOVO-Shcon groups compared to the Sh groups upon LPS stimulation, the immunofluorescence results also showed that the levels of acetyl-K29-HMGB1 were decreased after MSI2 knockdown (Fig. 4F-G). Consistent with this finding, in the nuclear-cytoplasmic fractionation assay, the cytoplasmic translocation and nuclear expression of acetyl-K29-HMGB1 were also found to be decreased in both the presence and absence of LPS stimulation after MSI2 knockdown (Fig. 4H-I). Furthermore, we investigated the effects of acetylation on the protein stability of HMGB1 and acetyl-K29-HMGB1. Treatment with the histone deacetylase inhibitors trichostatin A (TSA) and/or nicotinamide (NAM) further increased the protein levels of HMGB1 and acetyl-K29-HMGB1 in HEK 293 T, SW620 and LOVO cells (Fig. 4J). Notably, immunoprecipitation with anti-HMGB1 and anti-acetyllysine antibodies in stable SW620 and LOVO cell lines further verified that MSI2 increased the acetylation of HMGB1 K29 residue (Fig. 4K). Based on these results, MSI2 might function to increase the acetylation of HMGB1 K29 residue.

Various KATs and KDACs have been reported to regulate non-histone protein acetylation in immune and metabolic networks [32, 37–39]. We next sought to determine which KATs or KDACs are regulated by MSI2 to affect the acetylation of K29 in HMGB1. We first analyzed the relationships between MSI2 expression and KAT or KDAC expression in the TCGA CRC database, and positive correlations were found between MSI2 expression and the expression of these KATs and KDACs (Fig. 4L and Fig. S8A). Then, we found by acetylomics that EP300 (P300), HDAC1 and HDAC2 were downregulated after MSI2 knockdown in stable SW620 cells, and this finding was confirmed by qRT–PCR (Fig. 4M and Fig. S8B). However, only EP300 was downregulated in the SW620-Sh and LOVO-Sh groups upon LPS stimulation (Fig. 4N). The EP300/YY1/P65 complex has been identified as a regulator of target gene expression [52], and P300, the key acetyltransferase family member, has been confirmed to participate in the acetylation of various histones and nonhistone proteins [32, 53, 54], and P300 has also been identified to interact directly with HMGB1 to regulate the PTMs [55]. More importantly, MSI2 was predicted to interact with the EP300/YY1 complex in the RAID Database, and this interaction was verified by shotgun LC–MS/MS transcription factor analysis of stable HT29 cells (Fig. S8C-D). Endogenous Co-immunoprecipitation (Co-IP) and immunofluorescence assays further confirmed the cytoplasmic interaction of MSI2 with EP300 in SW620 and LOVO cells (Fig. 4O-P). And acetyl-K29-HMGB1 levels were also obviously reduced in the extracellular supernatant of stable SW620 and LOVO Shcon group cells treated with acetyltransferase P300 inhibitor (C646) compared to untreated cells (Fig. S8E). In addition, the protein levels of P300, HMGB1 and K29-HMGB1 and NF-κB signaling pathway components in Msi2^*Transgenic*^ CAC mice and stable CRC cell lines were also examined, and MSI2 was found to increase the protein levels of P300, HMGB1, K29-HMGB1 and p-p65 (Fig. 4Q-R and Fig. S8F). In conclusion, these findings suggest that MSI2 interacts with the cytoplasmic acetyltransferase P300 to promote K29-HMGB1 acetylation, translocation and extracellular release.

### MSI2 directly increases HMGB1 translation by binding to its 3’UTR

**Fig. 5:**
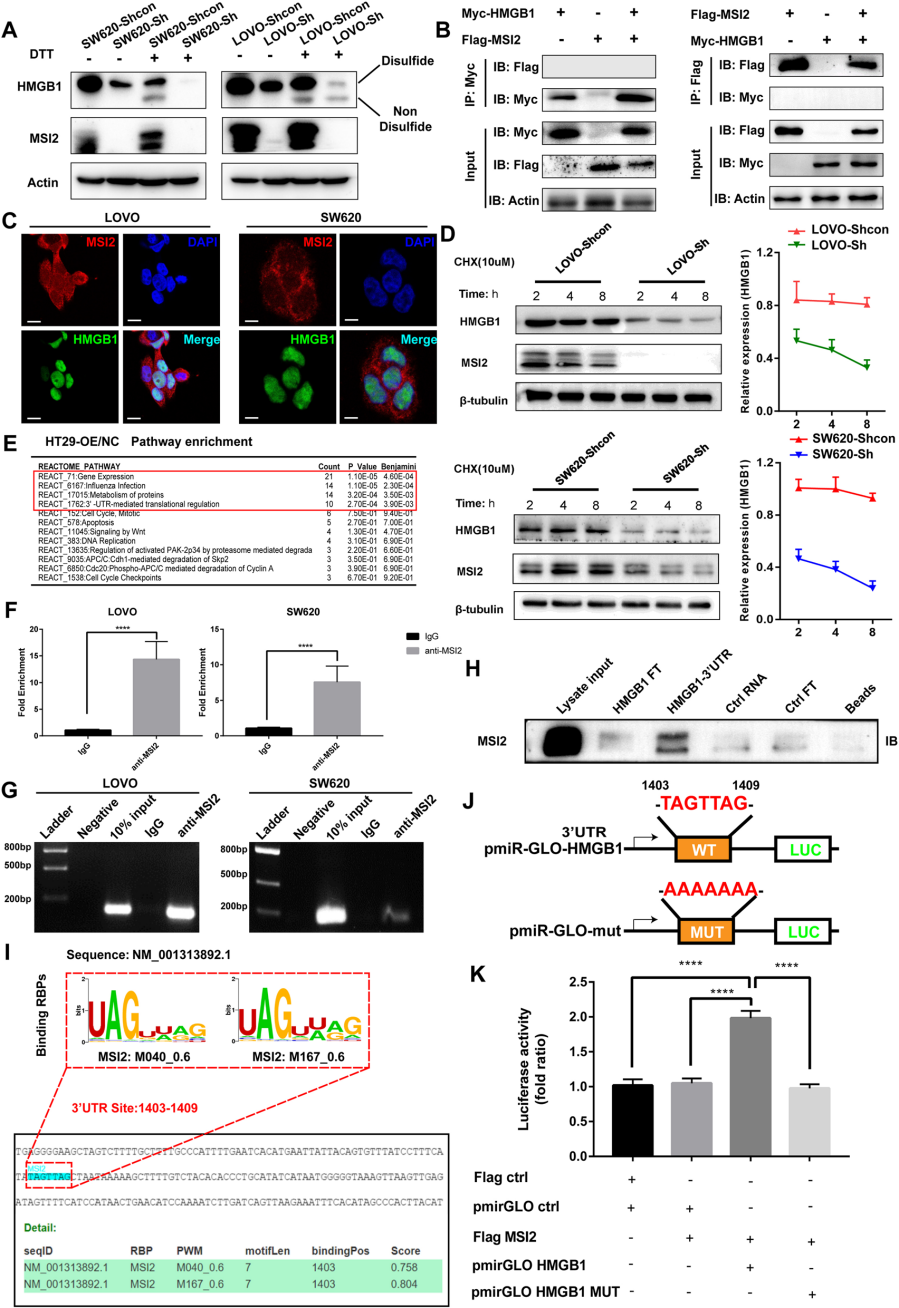
MSI2 directly increases disulfide HMGB1 production and translation by binding to nucleotides 1403–1409 in the 3’UTR of HMGB1. **A** Western blot analysis of disulfide and non-disulfide HMGB1 in lysates from stable SW620 and LOVO cells treated with or without DTT, and the Recombinant-HMGB1 (1 μg) incubated with either H_2_O_2_ or DTT (1 and 5 mM) were used as controls. **B** Exogenous Co-immunoprecipitation of Flag-MSI2 and Myc-HMGB1 in HEK 293 T cells. **C** Representative IFC images of MSI2 and HMGB1 localization in SW620 and LOVO cells. Scale bars, 20 μm. **D** MSI2 and HMGB1 protein levels in stable SW620 and LOVO cells at different time points after treatment with CHX (10 μM). **E** Reactome pathway enrichment analysis of the differentially expressed proteins identified by shotgun LC–MS/MS in HT29-OE/NC cells using the DAVID bioinformatics tool. **F** RIP assays were performed in LOVO and SW620 cells (anti-MSI2 and anti-IgG). **G** Detection of the HMGB1 gene by electrophoresis on a 2% agarose gel after RIP and qRT–PCR. **H** RNA pull-down assay was performed in LOVO cells, with Flowthrough supernatant (FT), biotinylated beads and lysate input as controls. **I** MSI2-specific RRMs predicted with beRBP: UAGUUAG. Binding scores (0.758 and 0.804) and putative positions of nucleotides 1403–1409 in the 3’UTR of HMGB1. **J** Wild-type (TAGTTAG) and mutant (AAAAAA) 3’UTRs of HMGB1 were inserted into the pmiR-GLO plasmid. **K** Firefly luciferase activity was measured in HEK 293 T cells transfected with MSI2-Flag and the wild-type or mutant pmiR-GLO plasmid and normalized to Renilla luciferase activity. These results are presented as the mean ± SD values; ****p < 0.0001; **F** unpaired 2-tailed Student’s t test and **K** one-way ANOVA

HMGB1 exists in multiple isoforms, including oxidized HMGB1, reduced HMGB1, sulfonyl HMGB1 and disulfide HMGB1, and these different isoforms play distinct functional roles in the tumor immune microenvironment [26–30]. To determine which HMGB1 isoforms are regulated by MSI2, lysates of stable SW620 and LOVO cells were treated with dithiothreitol (DTT), a strong reducing agent capable of dissociating disulfide bonds [56], and we found that the secondary non-disulfide isoforms of HMGB1 were emerged and that MSI2 mainly promoted the production of the disulfide isoform of HMGB1 compared with the controls in Recombinant-HMGB1 incubated with either H_2_O_2_ or DTT (Fig. 5A). Consistent with this finding, previous study also demonstrated that the disulfide HMGB1 was important for the maturation of DCs and immune infiltration in the TIME [28]. In addition to the aberrant cytoplasmic acetylation and translocation, nuclear HMGB1 levels were also significantly decreased after MSI2 knockdown, as shown in Figs. 3F and G, Fig. 4H and I. Thus, we sought to explore is the possibility of a direct interaction between MSI2 and HMGB1 by exogenous Co-immunoprecipitation and immunofluorescence assays and found no direct interaction at the protein level (Fig. 5B-C). MSI2 was also found to be involved in regulating protein translation, as shown in Fig. 4D and Fig. S7D and previous study [41]. Therefore, we further treated stable SW620 and LOVO cells with the protein synthesis inhibitor cycloheximide (CHX), and the Western blot results indicated that MSI2 can regulate the protein half-life of HMGB1 and enhance its translation capacity (Fig. 5D). Reactome pathway enrichment analysis of shotgun LC–MS/MS data for HT29 OE/NC cells also indicated the dysregulation of pathway including 3′-UTR-mediated translational regulation, influenza infection, metabolism of proteins and gene expression (Fig. 5E). Additionally, we conducted RNA immunoprecipitation (RIP) assays to determine whether MSI2 directly binds to the HMGB1 3’UTR, as evidenced by the qRT–PCR and gel electrophoresis results (Fig. 5F-G). Consistent with this finding, the direct association between the HMGB1 3’UTR and the MSI2 protein was also verified by RNA pulldown assay (Fig. 5H). Subsequently, Binding Estimation for human RNA-Binding Proteins (beRBP) predictions (http://bioinfo.vanderbilt.edu/beRBP/predict.html) identified UAGUUAG and UAGUAAG as the 2 important RNA recognition motifs (RRMs) in the MSI2 protein. We analyzed the HMGB1 3’UTR sequence and found that the putative binding site TAGTTAG had high binding scores (0.758 and 0.804) for interaction with nucleotides 1403–1409 in the HMGB1 3’UTR (Fig. 5I). Finally, the pmiR-GLO luciferase reporter plasmids containing the wild-type or mutated HMGB1 3’UTR and the MSI2-Flag plasmid were cotransfected into HEK 293 T cells, and luciferase reporter assays demonstrated the direct association between nucleotides 1403–1409 in the HMGB1 3′-UTR and MSI2 (Fig. 5J-K). Collectively, these data reveal that MSI2 enhances the more disulfide HMGB1 production and translation by directly binding to nucleotides 1403–1409 in the 3’UTR of HMGB1.

### Blockade of HMGB1 attenuates MSI2-mediated immunopathology in CRC

**Fig. 6:**
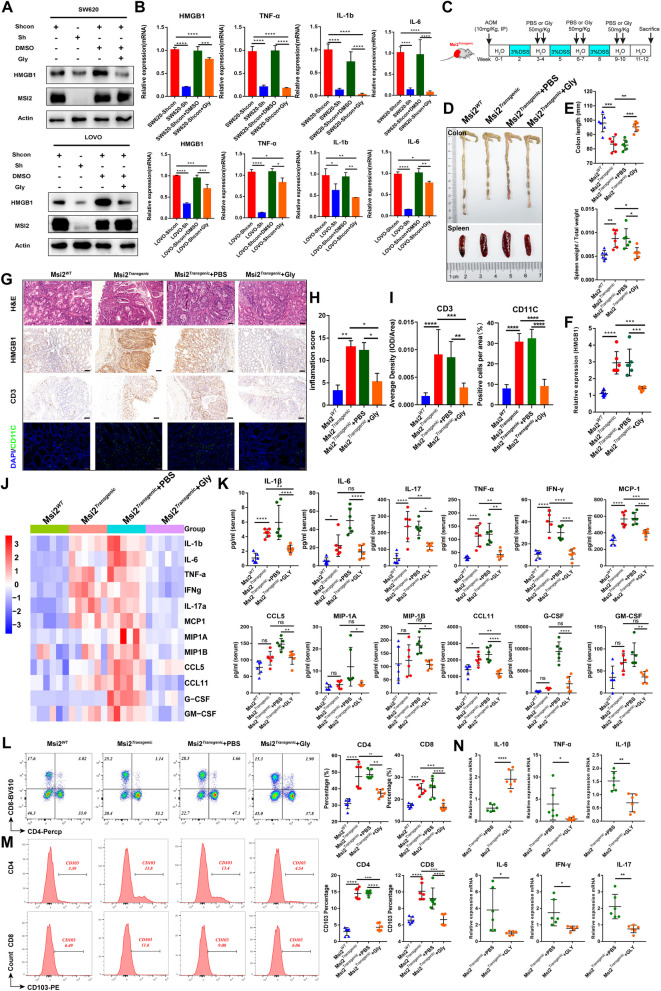
HMGB1 blockade by Gly attenuates MSI2-mediated CRC immunopathology in vitro and in vivo. **A-B** Western blot analysis of HMGB1 protein expression and qRT–PCR analysis of HMGB1, IL-6, TNF-a and IL-1b mRNA expression in stable SW620 and LOVO cells treated with DMSO or Gly (1 μM) for 12 h. **C** Diagram of the Gly (50 mg/kg) or PBS treatment protocol for CAC Msi2^*Transgenic*^ mice. **D** Representative colon and spleen images of Gly blockade CAC models. Colon (top) and spleen (bottom). **E** Statistical analysis of colon length and spleen weight in Gly-treated CAC mice, *n* = 6. **F** HMGB1 mRNA expression was measured by qRT–PCR in Gly-treated CAC mice, *n* = 6. **G** Representative H&E, IHC and IFC images of HMGB1, CD3 and CD11c staining in Gly-treated CAC mice. Scale bars, 100 μm. **H** Inflammation score analysis for Gly-treated CAC mice, *n* = 6. **I** Statistical analysis of the CD3 average IHC staining density and the proportions of CD11c-positive cells. **J** Heatmap of different cytokines and chemokines levels in serum using Bio-Plex Pro Mouse Cytokine 23-Plex immunoassay from Gly-treated CAC mice, *n* = 6. **K** Statistical analysis of serum cytokines and chemokines in Gly-treated CAC mice, *n* = 6. **L** FACS and statistical analysis of the percentages of CD4^+^ T cells and CD8^+^ T cells isolated from MLNs of Gly- or PBS-treated CAC mice, *n* = 6. **M** FACS and statistical analysis of the percentages of CD4^+^ CD103^+^ T cells and CD8^+^CD103^+^ T cells isolated from MLNs of Gly- or PBS-treated CAC mice, *n* = 6. **N** The mRNA levels of colonic inflammation-associated genes were measured by qRT–PCR in Gly-treated CAC mice (*n* = 6). These results are presented as the mean ± SD values; *p < 0.05, **p < 0.01, ***p < 0.001, ****p < 0.0001; **B, E-F, H-I, K-M** one-way ANOVA and **N** unpaired 2-tailed Student’s t test

Glycyrrhizic acid (Gly), a known antagonist of HMGB1, can trap the HMGB1 protein and thus blocks the extracellular release and cytoplasmic translocation of HMGB1, then significantly decrease the tissue inflammation and immune cell recruitment [57–60]. To investigate whether HMGB1 released from CRC tumor cells is responsible for MSI2-mediated immune activation in vitro and in vivo, we first treated stable SW620 and LOVO cells with Gly or DMSO for 24 h. The Western blot and qRT–PCR results indicated that Gly inhibited the production of inflammatory factors, including TNF-α, IL-1β and IL-6 (Fig. 6A-B). Then, we established a model of AOM/DSS-induced CAC with simultaneous intraperitoneal injection of PBS or Gly in Msi2^*Transgenic*^ mice (Fig. 6C). After blockade by Gly in Msi2^*Transgenic*^ mice, the colon length was significantly longer and the spleen size was significantly smaller than the PBS group (Fig. 6D-E). Meanwhile, in Gly-treated Msi2^*Transgenic*^ mice, the expression of HMGB1 was significantly downregulated (Fig. 6F-G), the inflammation score was decreased (Fig. 6H), and the proportions of CD3^+^ T-cell infiltration and CD11c-positive cells were also reduced, as evidenced by IHC and IFC staining (Fig. 6G and I). Moreover, the serum levels of the inflammatory cytokines IL-1β, TNF-α, IL-6, IFN-γ and IL-17a and the chemokines MCP-1, CCL5, CCL11, G-CSF and GM-CSF were significantly decreased in the Gly blockade group compared with the PBS group (Fig. 6J-K). Additionally, we further analyzed the infiltrated immune cells isolated from Msi2^*Transgenic*^ CAC mice treated with Gly or PBS by FACS and found that the percentages of the CD4^+^, CD8^+^ and CD11b^+^CD11c^+^ cell populations were decreased in Msi2^*Transgenic*^ CAC mice after Gly blockade (Fig. 6L and Fig. S9A). The migration of immune cells, including CD4^+^CD103^+^ T cells, CD8^+^CD103^+^ T cells and CD11b^+^CD11c^+^CD103^+^ cells, was also reduced after Gly blockade (Fig. 6M and Fig. S9B). Consistent with above finding, decreased mRNA levels of colonic inflammatory cytokines such as IL-1β, TNF-α, IL-6, IFN-γ, and IL-17a and increased mRNA level of IL-10 were found in Gly-treated Msi2^*Transgenic*^ CAC mice (Fig. 6N). Taken together, these results reveal the importance of HMGB1 blockade by Gly in attenuating MSI2-mediated CRC immunopathology in vitro and in vivo.

### Clinical effect of MSI2 on immune infiltration in CRC TIME

**Fig. 7:**
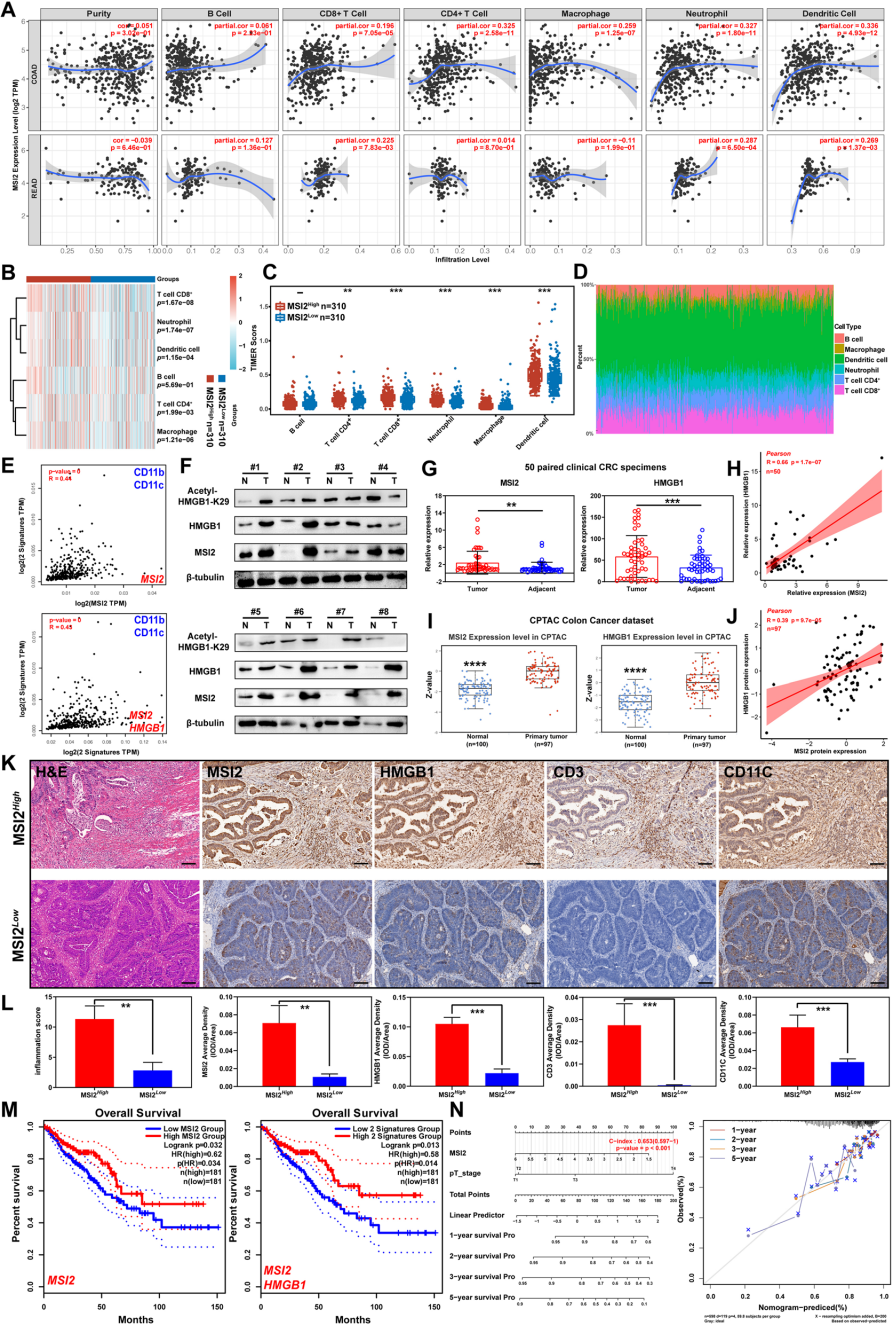
MSI2 regulates the TIME in clinical CRC specimens via HMGB1-mediated DC infiltration. **A** Positive *Spearman* correlations between MSI2 expression and the abundances of infiltrated immune cell types, including B cells, CD8^+^ T cells, CD4^+^ T cells, macrophages, neutrophils and DCs, in the COAD and READ datasets from TIMER database. **B** Immune cell score heatmap, different colors represent different MSI2 expression distribution in MSI2-high (*n* = 310) and MSI2-low (*n* = 310) CRC patients, the expression distribution of immune score were used immuneeconv-TIMER. **C** Statistical analysis of TIMER scores of B cells, CD8^+^ T cells, CD4^+^ T cells, macrophages, neutrophils and DCs in MSI2-high (*n* = 310) and MSI2-low (*n* = 310) CRC patients. **D** The percentage abundance of tumor infiltrating immune cells in each CRC specimens from TCGA (*n* = 620). Different colors indicate the different types of immune cells. **E** Positive *Spearman* correlations between MSI2 or (and) HMGB1, CD11b and CD11c expression in GEPIA CRC datasets. **F** Western blot analysis of MSI2, HMGB1 and K29-HMGB1 expression in 8 paired clinical CRC specimens. **G** Relative MSI2 and HMGB1 expression in 50 paired clinical CRC specimens. **H** *Pearson* correlation coefficients analysis showed a strong positive association between MSI2 and HMGB1 expression in our 50 paired clinical CRC specimens (R = 0.66, *p* = 1.7e-07). **I** The protein expression levels of MSI2 and HMGB1 in normal and primary tumor tissues from CPTAC (Normal, *n* = 100; Primary tumor, *n* = 97) Colon Cancer dataset. **J **Positive *Pearson* correlation between MSI2 and HMGB1 protein expression in the CPTAC Colon Cancer datasets (R = 0.39, *p* = 9.7e-05). **K-L** Representative H&E and IHC images of MSI2, HMGB1, CD3, and CD11c staining, histological inflammation score and statistical analysis of the average IHC staining density in MSI2-high and MSI2-low clinical CRC patient tissues. Scale bars, 100 μm. **M** Lower MSI2 or (and) HMGB1 expression predicted a poorer overall survival prognosis in GEPIA CRC datasets. The cutoff value was set at 50% with normalization to ACTB and analysis by the log-rank test, *n* = 362. **N** Nomogram prediction of 1-year, 2-year, 3-year and 5-year overall survival of TCGA CRC patients based on MSI2 expression. The calibration curve for the overall survival nomogram model in each discovery group and the dashed diagonal line represents the ideal nomogram. These results are presented as the mean ± SD values; **p < 0.01, ***p < 0.001, ****p < 0.0001; **B-C, I** Wilcoxon test, **G** paired 2-tailed Student’s t test and **L** Mann–Whitney test

To determine whether MSI2 is involved in immune infiltration in clinical CRC specimens, the correlations between MSI2 expression and immune infiltration were first examined in the COAD and READ datasets via the TIMER database. Notably, the expression of MSI2 was positively correlated with the infiltration levels of DCs (COAD, *R* = 0.336; READ, *R* = 0.269), neutrophils (COAD, *R* = 0.327; READ, *R* = 0.287), macrophages (COAD, *R* = 0.259), CD4^+^ cells (COAD, *R* = 0.325) and CD8^+^ T cells (COAD, *R* = 0.196; READ, *R* = 0.225) (Fig. 7A). Consistent with this finding, the TIMER scores were significantly higher in CD4^+^ T cells (*p* = 1.99e-03), CD8^+^ T cells (*p* = 1.67e-08), DCs (*p* = 1.15e-04), neutrophils (*p* = 1.74e-07) and macrophages (*p* = 1.21e-06) in patients with high MSI2 expression compared with the low expression group (Fig. 7B-D). The positive associations of MSI2 or (and) HMGB1 expression with the DC markers CD11b and CD11c in CRC were further determined in GEPIA CRC datasets (*R* = 0.44, *p* = 0; *R* = 0.43, *p* = 0) (Fig. 7E). In addition, the protein levels of MSI2 and HMGB1 were increased in primary CRC specimens compared with normal adjacent tissues in our clinical CRC specimens and CPTAC Colon cancer dataset (Fig. 7F-G and I). Remarkably, the protein expression levels of HMGB1 and acetyl-K29-HMGB1 were increased in CRC tissues with the upregulation of MSI2 (Fig. 7F), and the protein expression levels of MSI2 and HMGB1 were highly positively correlated in our clinical CRC specimens (*R* = 0.66, *p* = 1.7e-07) (Fig. 7H) and CPTAC Colon cancer dataset (*R* = 0.39, *p* = 9.7e-05) (Fig. 7J). Moreover, histological evaluation of our clinical CRC specimens showed that patients with high MSI2 expression also had higher inflammation scores and higher protein expression levels of HMGB1, CD3 and CD11c, as well as higher levels of CD11b, CD68 and Ly6g (Fig. 7K-L and Fig. S10A). Finally, we analyzed the relationship between MSI2 expression and prognosis, the overall survival (OS) analysis based on RNA FPKM values from TCGA showed that CRC patients with higher expression of MSI2 had a better prognosis (3 groups, *p* = 0.051; 4 groups, *p* = 0.015) (Fig. S10B). Importantly, the univariate and multivariate Cox regression analyses indicated that MSI2 was a good independent factor for 5-year overall survival (Uni-Cox, HR = 0.73097, *p* = 0.02952; Mult-Cox, HR = 0.59168, *p* = 0.00937) (Fig. S10C). Moreover, the patients with higher expression of MSI2 or (and) HMGB1 also had a longer overall survival in GEPIA CRC datasets (HR = 0.62, Log rank *p* = 0.032; HR = 0.58, Log rank *p* = 0.013) (Fig. 7M). The Nomogram prediction of 1-year, 2-year, 3-year and 5-year overall survival in TCGA CRC patients also showed that higher MSI2 expression was associated with a better prognosis (Fig. 7N). Collectively, these results indicate that MSI2 improves tumor immunity in CRC and that better patient outcomes may be due to HMGB1-mediated immune infiltration.

## Discussion

CRC is one of the three most common malignancies worldwide, and combination treatment with current neoadjuvant therapies and immunotherapy has improved patient survival. Thus, identifying important crosstalk between tumor cells and infiltrating immune cells in the CRC TIME is essential for the efficacy of immunotherapy. This study revealed a new pivotal role of MSI2 in regulating HMGB1 PTMs to influence immune infiltration and inflammatory responses in CRC.

Currently, data describing the effects of MSI2 on immune function are scarce, but a few articles have confirmed that MSI2 contributes to the inflammatory process and immune response [11–13, 16, 17]. However, the regulation of HMGB1 by RBPs is still largely uncharacterized, as is tumor immunometabolism. Through in vitro and in vivo assays, we observed that MSI2 can enhance immune infiltration and the inflammatory response in CRC, with effects such as increasing the proportions of DCs, CD4^+^ T cells and CD8^+^ T cells and the production and secretion of inflammatory factors, cytokines and chemokines. These results were further supported by analyses of GEO and TCGA datasets, and IHC analysis of our clinical specimens proved MSI2-related immune infiltration. Additionally, as demonstrated in previous reports [61, 62], we found that higher MSI2 and HMGB1 expression predicted a better overall survival prognosis, possibly because MSI2 might regulate the production, subcellular localization and release of HMGB1. We therefore performed further experiments to test this hypothesis. As shown in Fig. 2, we examined the differentially expressed proteins by proteomic analysis and the CAC model of colon cancer in transgenic mice with intestinal epithelial overexpression of MSI2 was established, MSI2 was found to increase the expression of HMGB1 in vitro and in vivo. Importantly, prior studies showed that HMGB1 is crucial for DC function and chemotaxis, which trigger inflammation [47, 48], and our coculture assay of human DCs with stable CRC cells further showed that MSI2 promotes HMGB1-mediated DCs maturation, as evidenced by the expression of DC maturation markers such as CD80, CD86, CD40 and CD83. Furthermore, our study in Msi2^*Transgenic*^ CAC mice also indicated that MSI2 contributes to immune infiltration via HMGB1-mediated immune cells migration, such as the upregulation of CD103 in DCs, CD4^+^ T and CD8^+^ T.

Despite these findings, the function and underlying mechanism of MSI2 in CRC immune infiltration are not well elucidated. Importantly, HMGB1 exerts different biological effects upon stimulation that are dependent mainly on its subcellular location, PTMs and redox state [23]. Previous studies have demonstrated that stress stimulates the translocation of HMGB1 from the nucleus to the cytoplasm and its subsequent release into the extracellular milieu to trigger proinflammatory cascades and recruit immune cells [24]. Next, we explored the subcellular localization of HMGB1 and the mechanisms that regulate this distribution. As shown in Fig. 3, we first found that MSI2 further increased the production, nucleocytoplasmic translocation and extracellular release of HMGB1 under LPS treatment, and MSI2 was also found to be involved in nucleocytoplasmic transport signaling based on KEGG pathway enrichment analysis of TCGA data from MSI2-high and MSI2-low patients. To determine how MSI2 regulates the subcellular translocation of HMGB1, we focused on the HMGB1 PTM process. The PTMs, such as acetylation, methylation, phosphorylation, ADP-ribosylation, lactylation and S-nitrosylation, that govern the cellular localization of HMGB1 have been demonstrated [31–34]. Chief among these PTMs is acetylation of critical lysine residues to regulate HMGB1 cytoplasmic accumulation [35, 36]. Through protein acetylome sequencing analysis, we identified two key residues of HMGB1 (K29 and K30) acetylated by MSI2, and further verification was performed based on the ectopic translocation of K29-HMGB1. In additional to this, to determine which KATs and KDACs play a key role in this PTMs, we performed TCGA database analysis, proteomic analysis and co-immunoprecipitation assays and clarified that MSI2 can interact with the cytoplasmic acetyltransferase P300 to upregulate its expression, further promoting HMGB1 acetylation at K29 and upregulating the NF-κB signaling pathway, thus leading to the translocation and extracellular release of K29-HMGB1.

More importantly, the redox state of HMGB1 plays distinct functional roles in the TIME. Previous evidence has indicated that the reduced, disulfide and thiol HMGB1 isoforms are considered proinflammatory cytokine-like or chemokine-like molecules that recruit inflammatory cells to promote immune activation and clearance. However, the oxidized HMGB1 isoform acts as an inducer to promote immune tolerance [26–30]. We therefore sought to determine which isoforms are regulated by MSI2 by using DTT to treat lysates of stable SW620 and LOVO cells and found that the secondary non-disulfide isoforms of HMGB1 were produced compared to the controls of Recombinant-HMGB1 incubated with H_2_O_2_ or DTT, suggesting that MSI2 mainly promoted the production of disulfide HMGB1, which is consistent with previous research results [28]. TLR4 and RAGE have been found to act as the major HMGB1 receptors, and disulfide HMGB1 can directly activate the TLR4 complex by binding to MD-2, thus triggering a series of inflammatory responses [63]. In addition to the aberrant cytoplasmic translocation of HMGB1, its nuclear level was also significantly decreased after MSI2 knockdown, and we suspect that MSI2 may be directly involved in affecting the translation of HMGB1, as shown in Fig. 4D (KEGG pathway analysis), Fig. S7D (COG/KOG pathway enrichment analysis) and previous study [41]. The results of further experimental verification suggested that MSI2 could enhance protein translation by directly binding to nucleotides 1403–1409 in the 3’UTR of HMGB1.

Given that considerable progress has been made in HMGB1-targeted therapy in multiple inflammatory conditions, especially in blocking the production of excessive amounts of the extracellular disulfide HMGB1 isoform, which provides constitutes an attractive clinical opportunity to ameliorate systemic inflammatory diseases [64], and extracellular HMGB1 blockade also controls tumor progression by remodeling the immune microenvironment [28]. Gly, an HMGB1 inhibitor, can trap and block the alarmin function of HMGB1 in the extracellular release and cytoplasmic translocation, thus significantly decreasing tissue inflammation and immune cell recruitment [59, 60, 65, 66]. After Gly treatment in Msi2^*Transgenic*^ CAC mice, HMGB1 blockade reduced immune infiltration within tumor tissues, as determined by findings such as the reductions in the migration and proportion of DCs, CD4^+^ T cells and CD8^+^ T cells and the production and secretion of inflammatory factors, cytokines and chemokines. These findings suggest that HMGB1 blockade by Gly can attenuate MSI2-mediated CRC immunopathology and immune infiltration. In addition, HMGB1 can mediate immunogenic cell death and enhance antitumor immunity [67]. On the one hand, HMGB1 can be rapidly released from dead and necrotic tumor cells as an alarmin upon treatment with chemotherapy or radiotherapy. On the other hand, extracellular HMGB1 can recruit inflammatory cells and mediate immune killing and clearance by promoting the maturation of antigen-presenting DCs and induction of immune attacks on CD8^+^ T cells. Therefore, elucidating the crosstalk between tumor-infiltrating immune cells and tumor cells will help to address the dilemma of tumor immunotherapy and shift the balance toward favorable instead of poor outcomes.

**Fig. 8:**
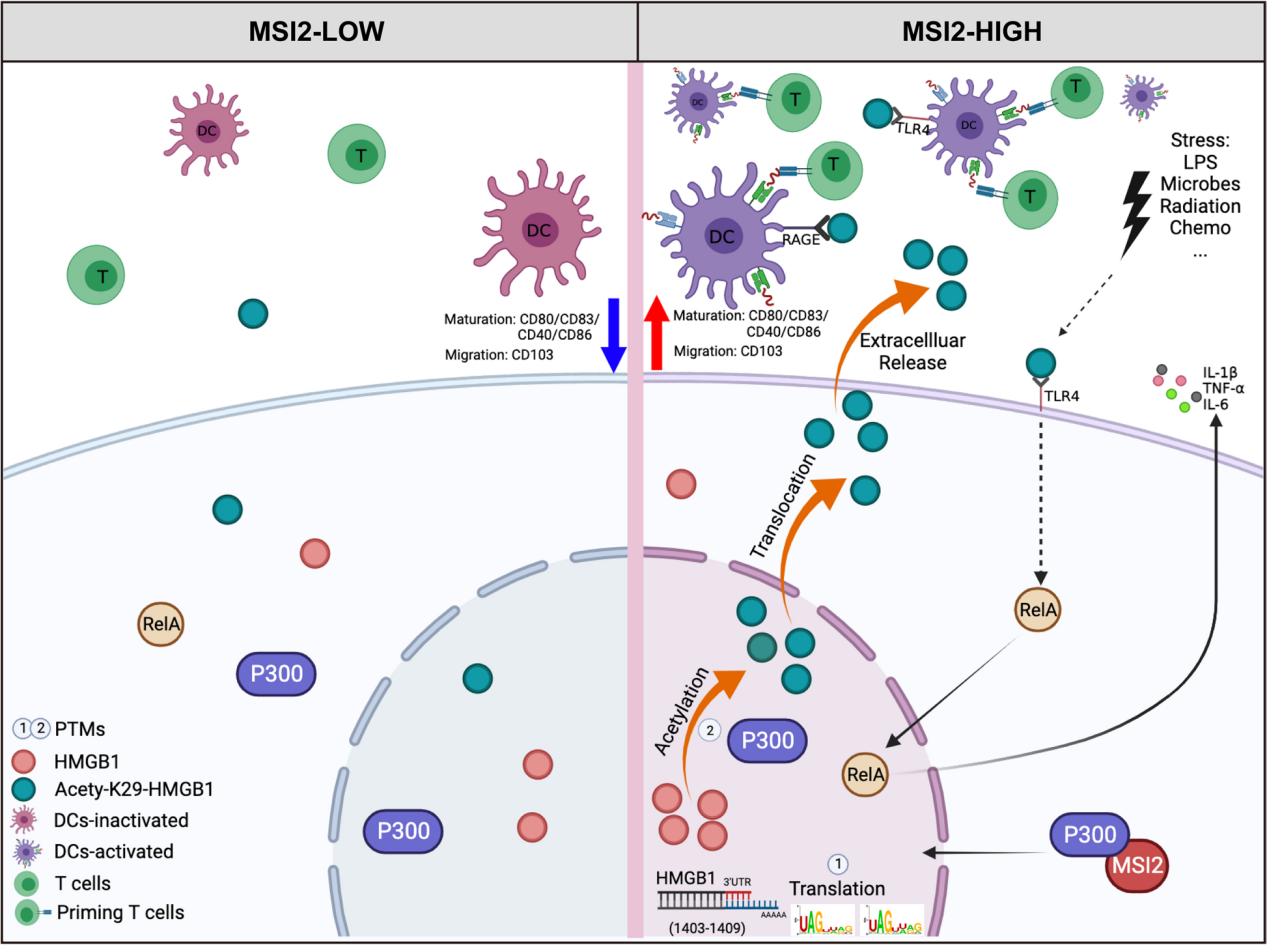
Schematic diagram of the present study. This study demonstrates that the MSI2 protein directly binds to nucleotides 1403–1409 in the 3’UTR of HMGB1 to increase disulfide HMGB1 production and translation and then interacts with the cytoplasmic acetyltransferase P300 to upregulate its expression, further promoting HMGB1 acetylation at K29 and the translocation and extracellular release of K29-HMGB1, leading to immune infiltration via HMGB1-mediated maturation and migration of DCs in CRC

Taken together, our work is the first to investigate the molecular mechanism of MSI2 in tumor immune infiltration and immunometabolism in CRC. MSI2 essentially promotes HMGB1 protein translation by directly binding to nucleotides 1403–1409 in the 3’UTR, affects the production of disulfide HMGB1, and then interacts with the cytoplasmic acetyltransferase P300 to upregulate its expression, further promoting K29 acetylation to increase K29-HMGB1 nucleocytoplasmic translocation and extracellular release. Ultimately, DCs, neutrophils, CD8^+^ T cells and other immune cells are recruited to the TIME for killing and immune clearance, which may improve the prognosis of CRC patients (Fig. 8). These findings elucidate the molecular mechanism by which MSI2 regulates the PTMs of HMGB1 to reshape the TIME and reprogram immunometabolism, providing new important insights into the role of MSI2 in CRC immunopathology.

### Supplementary Information

**Additional file 1.**

**Additional file 2.**

**Additional file 3.**

**Additional file 4.**

## Data Availability

No datasets were generated or analysed during the current study.

## Abbreviations

PTMs: post-translational modifications
HMGB1: high-mobility group protein B1
RBP: RNA-binding protein
CRC: colorectal cancer
CAC: colitis associated colon cancer
DCs: dendritic cells
DAMPs: damage-associated molecular patterns
Gly: Glycyrrhizin acid
TIME: tumor immune microenvironment
MLN: mesenteric lymph nodes
SP: spleen
CLP: colonic mucosal lamina propria
TCGA: The Cancer Genome Atlas
GEO: Gene Expression Omnibus
GO: Gene Ontology
TIMER: Tumor Immune Estimation Resource
KEGG: Kyoto Encyclopedia of Genes and Genomes
TSA: Trichostatin A
NAM: Nicotinamide
CHX: Cycloheximide
NLSs: nuclear localization sequences
KATs: lysine acetyltransferases
KDACs: lysine deacetylases
