# Supplementary material for: Musashi-2 potentiates colorectal cancer immune infiltration by regulating the post-translational modifications of HMGB1 to promote DCs maturation and migration
Source: Cell Commun Signal. 2024 Feb 12;22:117. doi: 10.1186/s12964-024-01495-z (PMC10863188; doi:10.1186/s12964-024-01495-z)
Supplement: Supplementary file 1 — Additional file 1. [file 12964_2024_1495_MOESM1_ESM.docx]

Supplementary Materials for

**Musashi-2 potentiates colorectal cancer immune infiltration by regulating the post-translational modifications of HMGB1 to promote DCs maturation and migration**

Xiaole Meng^1,2,3,4†^, Risi Na^1,4†^, Xiao Peng^4†^, Hui Li^3†^, Wanxin Ouyang^1,4^, Wenting Zhou^1,4^, Shuming Zhang^3^, Xuting You^1,3^, Yuhuan Li^1,3^, Xin Pu^1,3^, Ke Zhang^1,3^, Junjie Xia^1^, Jie Wang^1,4*^, Huamei Tang^1,3*^, Guohong Zhuang^1*^, Zhihai Peng^1,4*^

**Affiliations**

^1^ Organ Transplantation Institute of Xiamen University, Xiamen Human Organ Transplantation Quality Control Center, Xiamen Key Laboratory of Regeneration Medicine, Fujian Provincial Key Laboratory of Organ and Tissue Regeneration, School of Medicine, Xiamen University, Xiamen, 361102, Fujian, China

^2^ National Institute for Data Science in Health and Medicine, Xiamen University, Xiamen, 361102, Fujian, China

^3^ Department of Pathology, Xiang'an Hospital of Xiamen University, School of Medicine, Xiamen University, Xiamen, 361102, Fujian, China

^4^ Organ Transplantation Clinical Medical Center of Xiamen University, Department of General Surgery, Xiang'an Hospital of Xiamen University, School of Medicine, Xiamen University, Xiamen, 361102, Fujian, China

†These authors have contributed equally to this work.

Correspondence to:

Zhihai Peng ([pengzhihai1958@163.com](mailto:pengzhihai1958@163.com)); Guohong Zhuang ([zhgh@xmu.edu.cn](mailto:zhgh@xmu.edu.cn)); Huamei Tang ([tanghuamei2014@163.com](mailto:tanghuamei2014@163.com)); Jie Wang(wangjielj2012@163.com)

**This file includes:**

Supplementary Figures, S1 to S10

Supplementary Tables, S1 to S4

**Supplementary Figure**

**
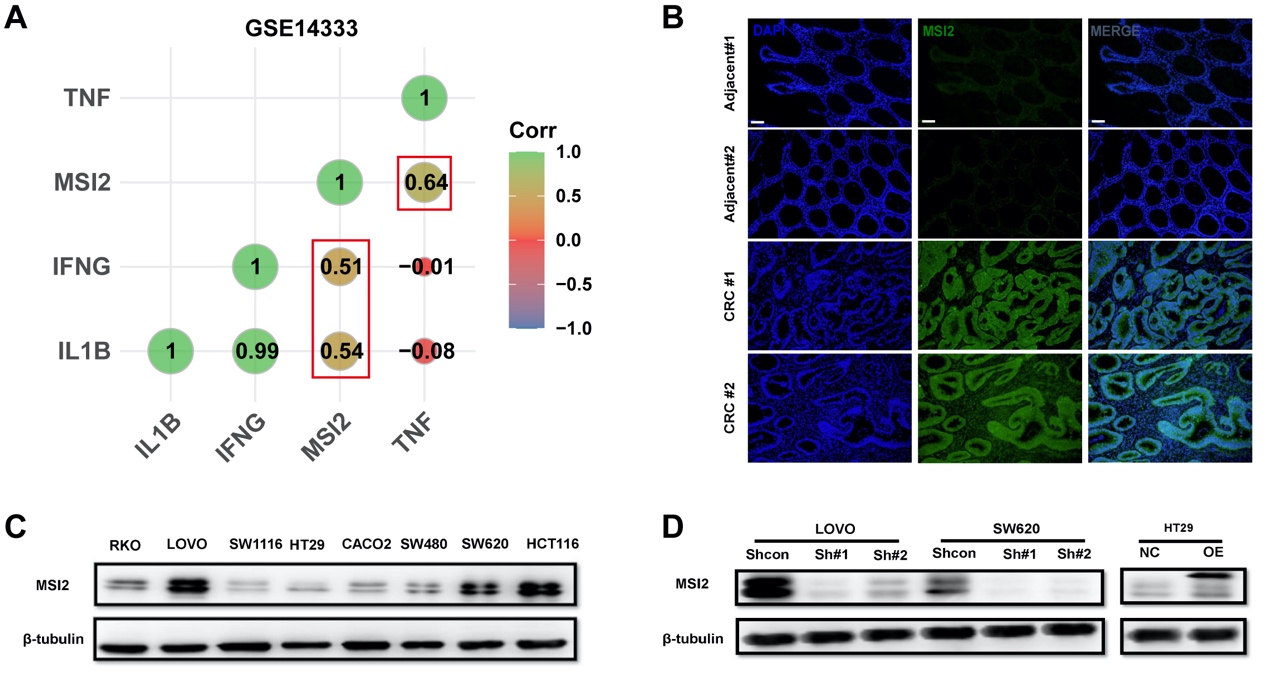
**

**Fig.S1**

**A**, Heatmap of the positive correlations between MSI2 and inflammatory-associated genes (TNF, IFNG and IL1B) in GSE14333 CRC database. **B**, Representative IFC images of MSI2 expression from clinical CRC and adjacent tissues. Scale bars, 100μm. **C**, Western blotting for MSI2 expression from CRC cell lines. **D**, Western blotting for MSI2 expression from SW620, LOVO and HT29 stable cell lines.


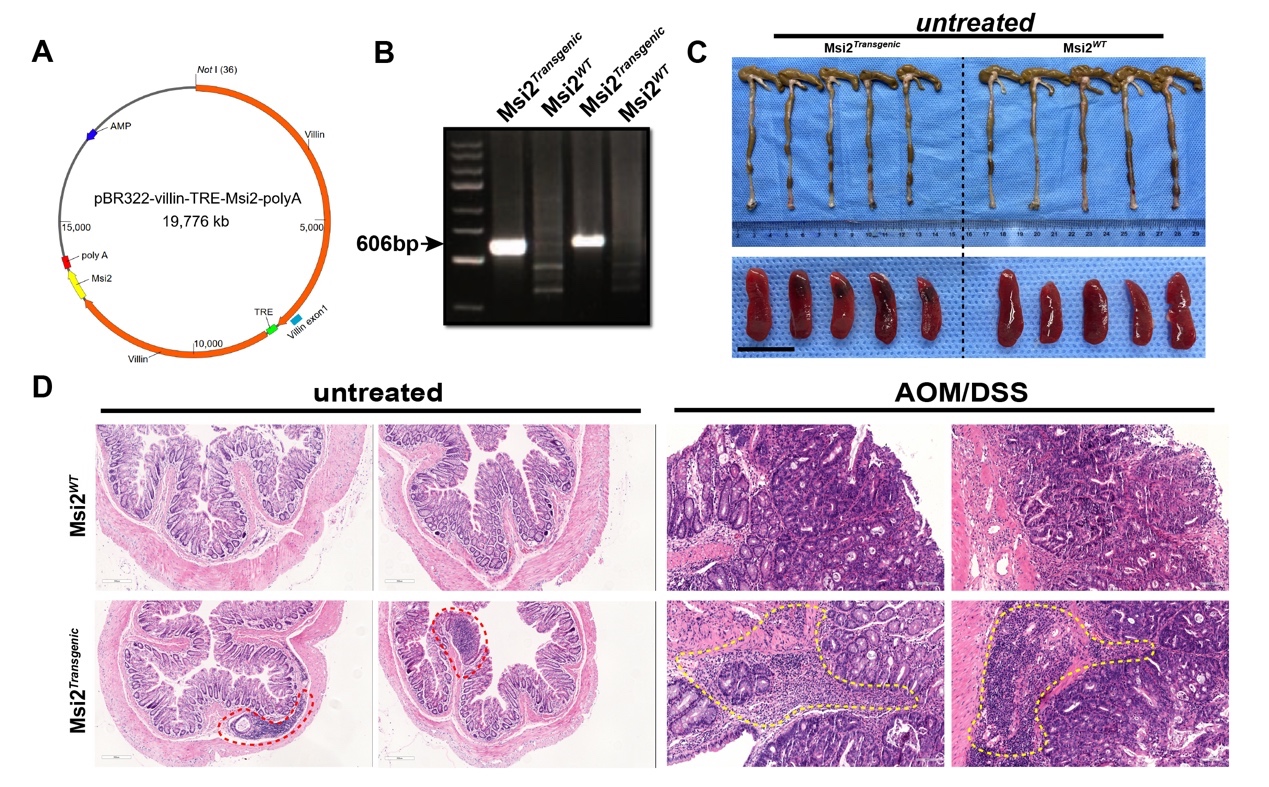


**Fig.S2**

**A**, The *Villin*-Msi2 transgenic mice (Msi2*^Transgenic^*) were generated using plasmids containing the mouse *Villin* promoter and Msi2 fusion gene. **B**, Genotyping of Msi2*^Transgenic^* mice identified by electrophoresis on a 2% agarose gel after PCR using tail DNA. **C,** Representative images of untreated Msi2*^Transgenic^* and WT mice. Colon (up) and spleen (bottom). Scale bar, 10mm. **D**, Representative colon images of H&E for untreated and AOM/DSS induced CAC mice, red dotted circles to highlight the immune infiltration in crypts lumen of untreated mice, and yellow dotted circles to highlight the immune infiltration in tumor stroma of CAC mice. Scale bars, 100μm.


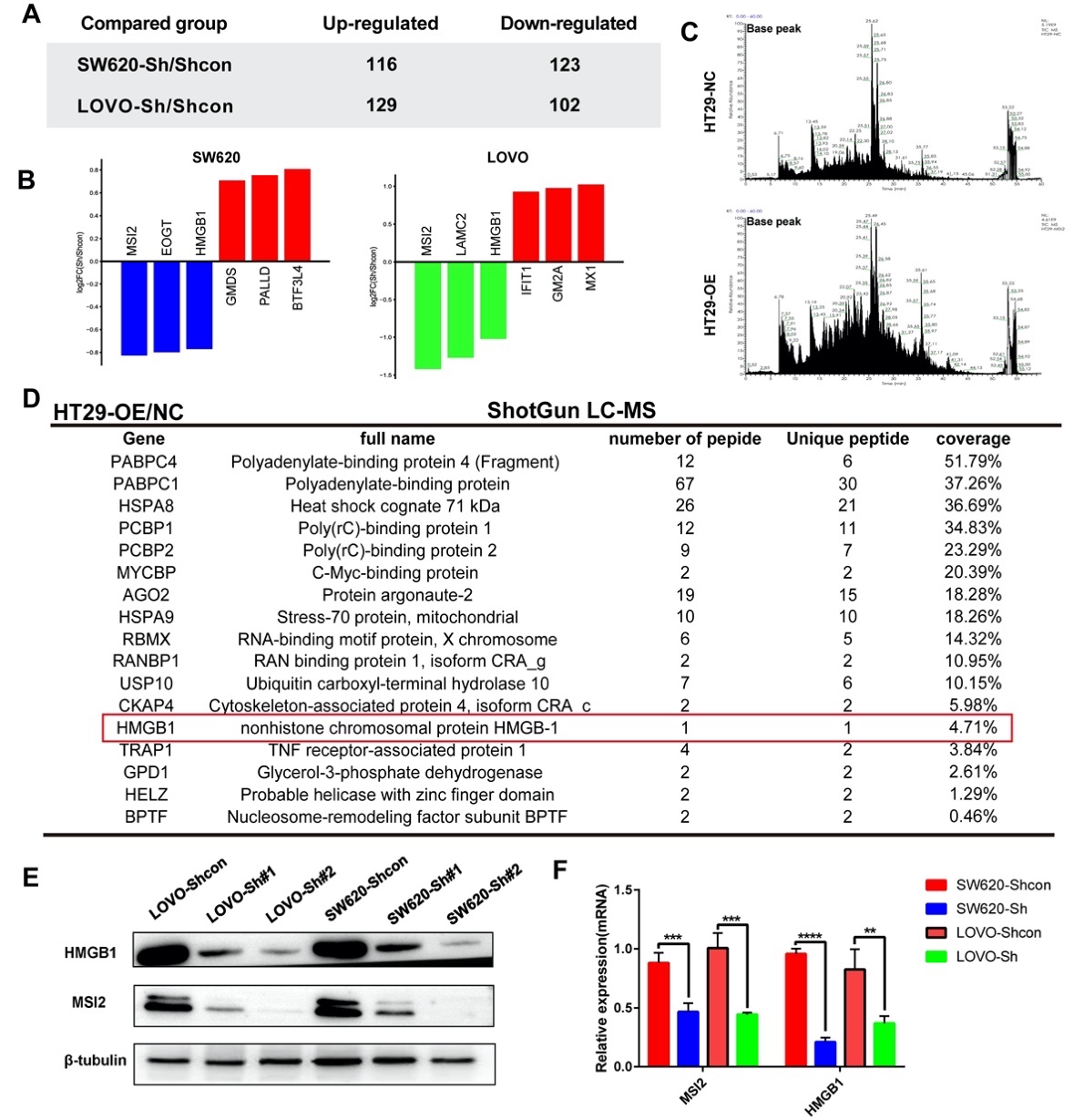


**Fig.S3**

**A-B**, The upregulated and downregulated protein genes from SW620 and LOVO stable cell lines proteomics analysis. **C**, Identification of HT29-NC and HT29-OE stable cells relative protein abundance by shotgun mass spectrometry. **D,** Partial list of differential proteins from shotgun LC-MS of HT29-NC/OE stable cells. **E**, Western blotting for MSI2 and HMGB1 expression in LOVO, SW620 stable cells. **F**, The MSI2 and HMGB1 mRNA expression levels were measured by qRT-PCR in LOVO, SW620 stable cells. These results are presented as the mean ± SD values; ***p*< 0.01, ****p*< 0.001, *****p*< 0.0001;(**F**) unpaired 2-tailed Student’s t-test.

**
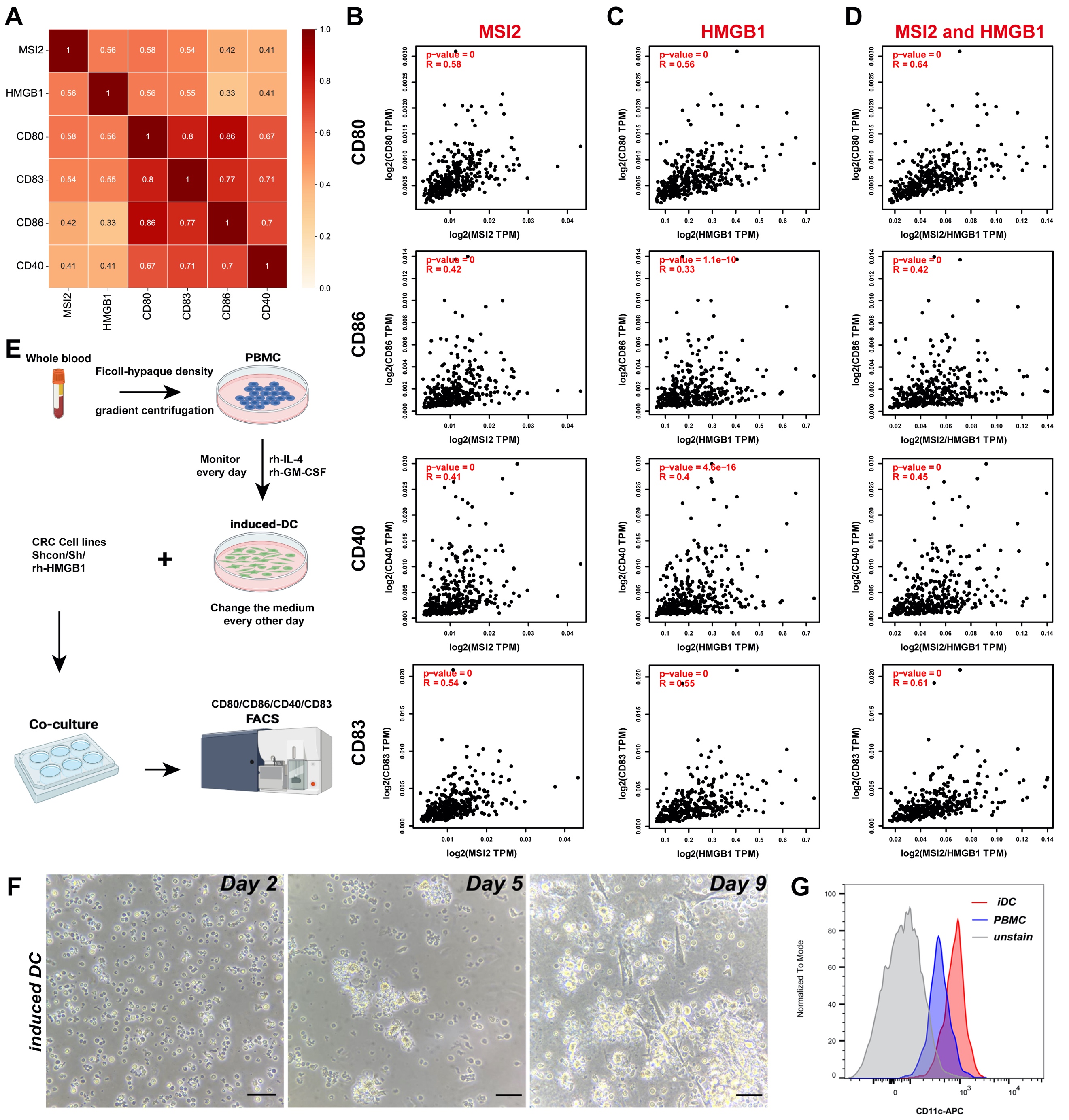
**

**Fig.S4**

**A**, Heatmap of the positive associations between MSI2, HMGB1 and DCs maturation markers (CD80, CD86, CD40, CD83) from GEPIA CRC database. **B-D**, The positive correlations between MSI2 or (and) HMGB1 and DCs maturation markers CD80, CD86, CD40, CD83 were identified by using *Spearman* correlation from GEPIA CRC database normalized by GAPDH, n=367. **E**, The flow diagram of induced DCs (iDCs) differentiation (healthy donor PBMC were treated with rh-IL-4 (40 ng/mL) and rh-GM-CSF (80 ng/mL) for 9 days) and co-culture with LOVO stable cells for 24h then for FACS analysis. **F**, Representative images of induced DCs (iDCs) at day2, day5 and day9. Scale bars, 100μm. **G**, Identification of iDCs by using FACS analysis of expression abundance of CD11c-APC.

**
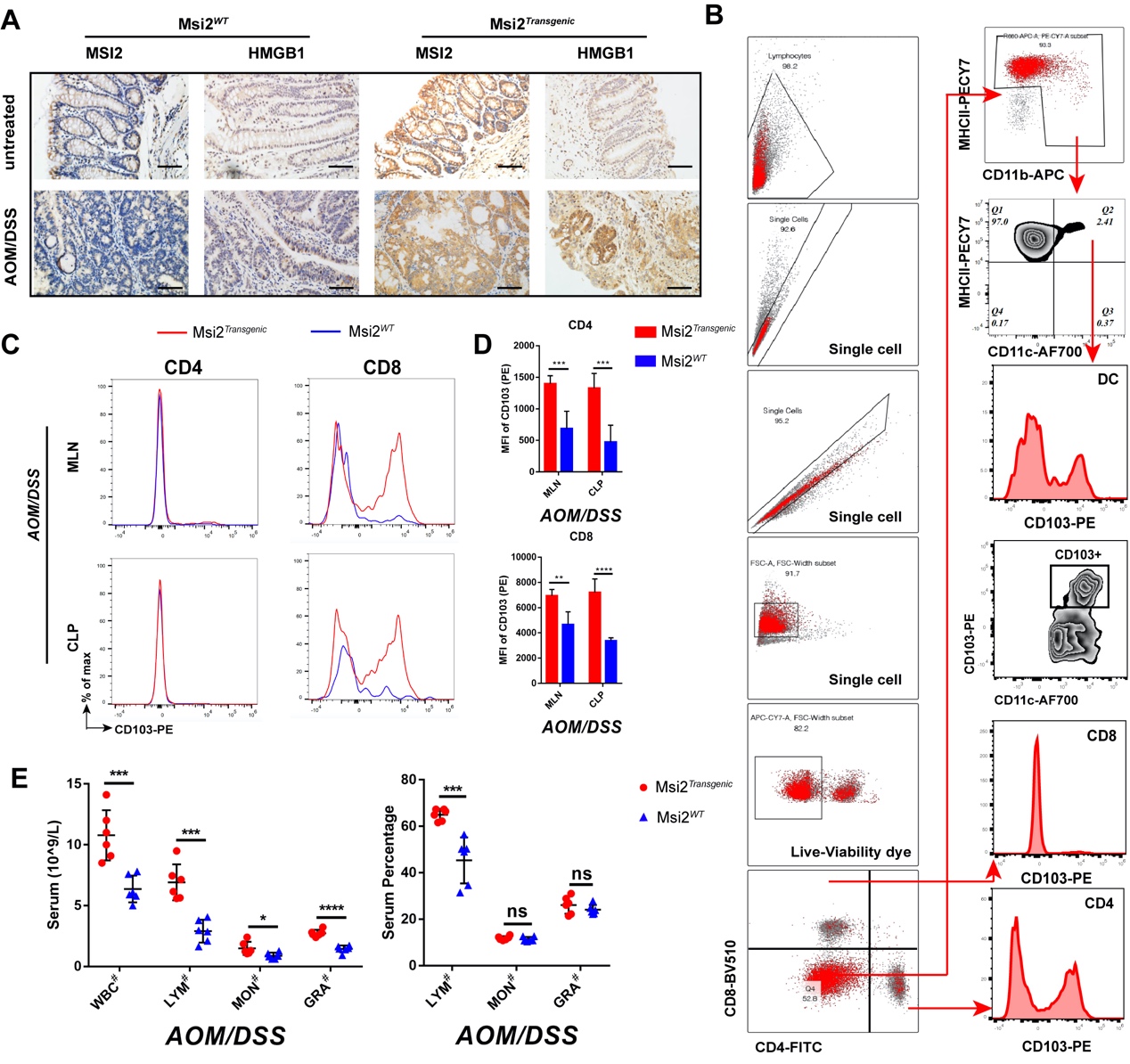
Fig.S5**

**A**, Representative IHC images of MSI2 and HMGB1 from untreated and CAC mice. Scale bars, 100μm. **B**, Flow cytometry gate strategy for the analysis of the CD4^+^, CD8^+^ T cells and DC cells isolated from mice Spleen, MLN and CLP. **C**, FACS analysis of CD103 expression with gating on CD4^+^ and CD8^+^ cells isolated from the MLNs and CLP of CAC mice. **D**, Statistical analysis of the CD103 mean fluorescence intensity (MFI) in CD4^+^ and CD8^+^ cells isolated from the MLNs and CLP of CAC (n=5) mice. **E**, Statistical analysis of the absolute number and proportion of white blood cells, lymphocytes, monocytes, and neutrophils in eyeball peripheral blood from CAC (n=6) mice. These results are presented as the mean ± SD values; ns: not significant, **p*< 0.05, ***p*< 0.01, ****p*< 0.001, *****p* < 0.0001; (**D-E**) unpaired 2-tailed Student’s t-test.

**
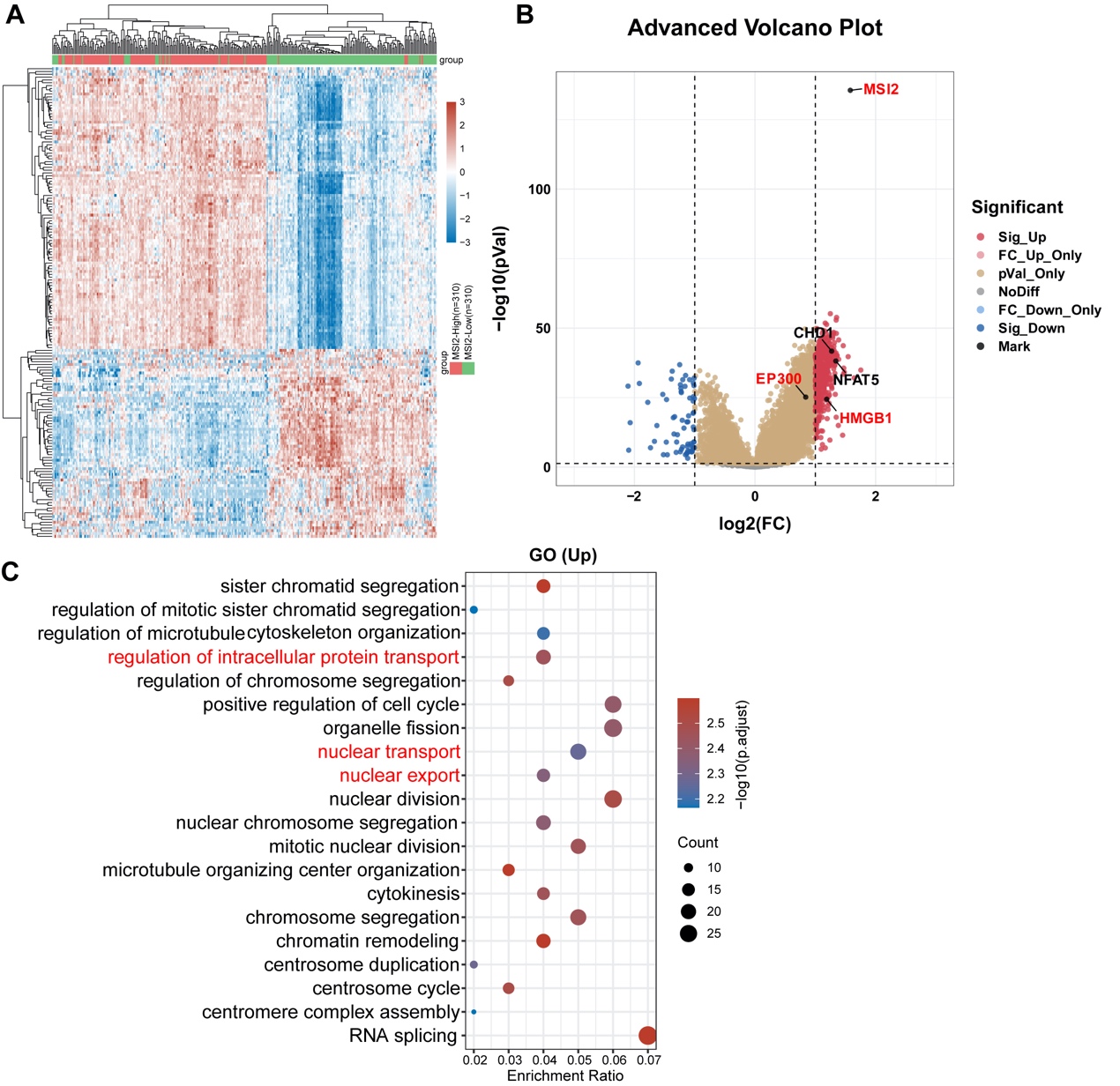
Fig.S6**

**A**, Heatmap of the differential gene expression in MSI2-high (n=310) and MSI2-low (n=310) patients from TCGA CRC database. **B**, The volcano plot of the differential gene expression in MSI2-high (n=310) and MSI2-low (n=310) patients RNA-Seq, “Adjusted p < 0.05 and Log2(Fold Change) >1.5 or < −1.5” were defined as the threshold for differential mRNA expression. Red dots indicate upregulated genes; blue dots indicate downregulated genes. **C**, The Functional enrichment Gene ontology (GO) analysis of potential targets of mRNAs. The up-regulated GO terms include regulation of intracellular protein transport, nuclear transport and nuclear export.


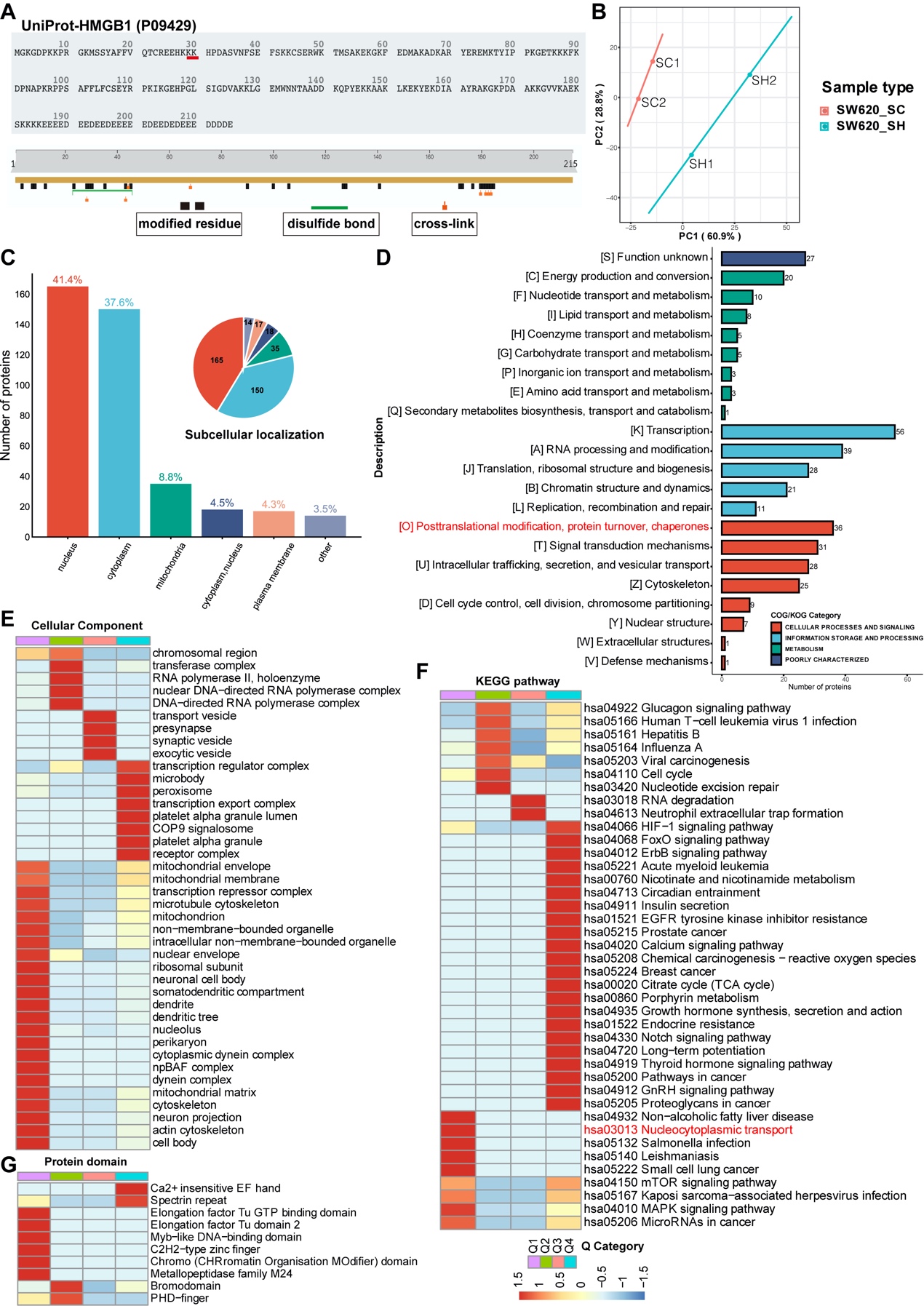


**Fig.S7**

**A**, The profiling of post-translation modification sites of HMGB1(P09429) from UniProt (https://www.uniprot.org/). **B**, The PCA acetylomics analysis of SW620-SC and SW620-SH stable cells. **C**, The cellular distribution characteristics of differential acetylation modified sites protein genes. **D**, The COG/KOG category of differential acetylation modified genes mainly enriched in Transcription, RNA processing and modification, posttranslational modification. **E**, The CC (cellular component) enrichment analysis of differential acetylation modified genes. **F**, The KEGG pathway enrichment analysis of differential acetylation modified genes, where Q4 clusters mainly enriched in Nucleocytoplasmic transport and Non-alcoholic fatty liver disease. **G**, The Protein domain enrichment analysis of differential acetylation modified genes mainly enriched in Myb-like DNA binding domain and C2H2-type zinc finger.


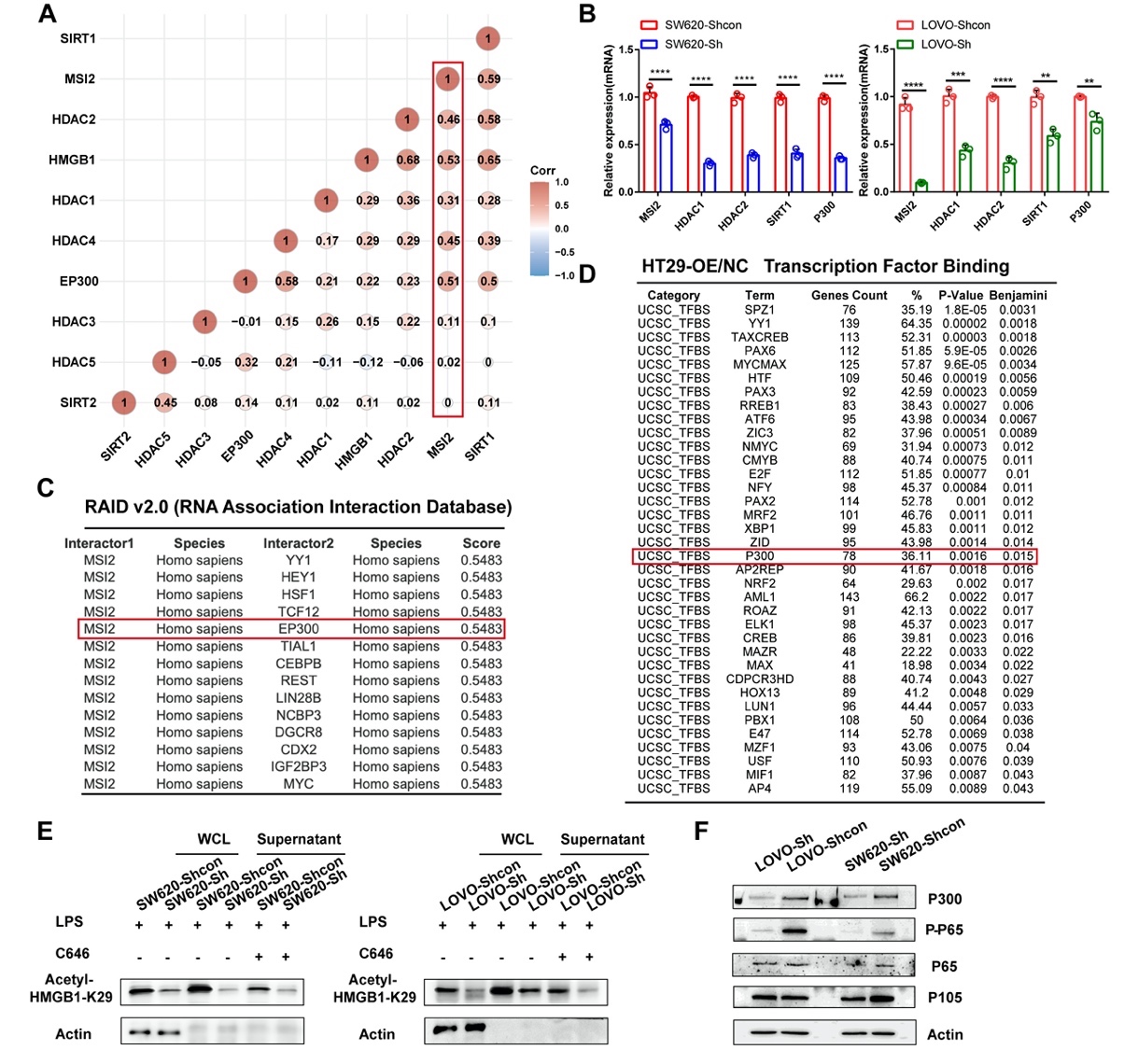


**Fig.S8**

**A**, Heatmap of the correlations between MSI2 and KATs or KDACs (such as HDAC1, HDAC2, HDAC3, HDAC4, HDAC5, SIRT1, SIRT2 and EP300) from TCGA CRC database. **B**, The MSI2, HDAC1, HDAC2, SIRT1 and EP300 mRNA expression were measured by qRT-PCR in LOVO, SW620 stable cells without LPS stimulation. **C**, The putative genes list that MSI2 could interact with from RAID v2.0 (<http://www.rna-society.org/raid2/index.html>), such as YY1, P300, LIN28B, MYC. **D**, The putative transcription factors binding genes list of differential proteins from shotgun LC-MS of HT29-OE/NC stable cells, such as YY1, P300. **E**, K29-HMGB1 expression in the whole cell lysate and extracellular supernatant of stable SW620 and LOVO cells treated with 10μg/mL LPS and acetyltransferase P300 inhibitor (C646) 10μM for 8 hours. **F**, Western blotting for P300, P65, p-P65 and P105 expression in LOVO, SW620 stable cells. These results are presented as the mean ± SD values; ***p* < 0.01, ****p* < 0.001, *****p* < 0.0001; (**B**) unpaired 2-tailed Student’s t-test.


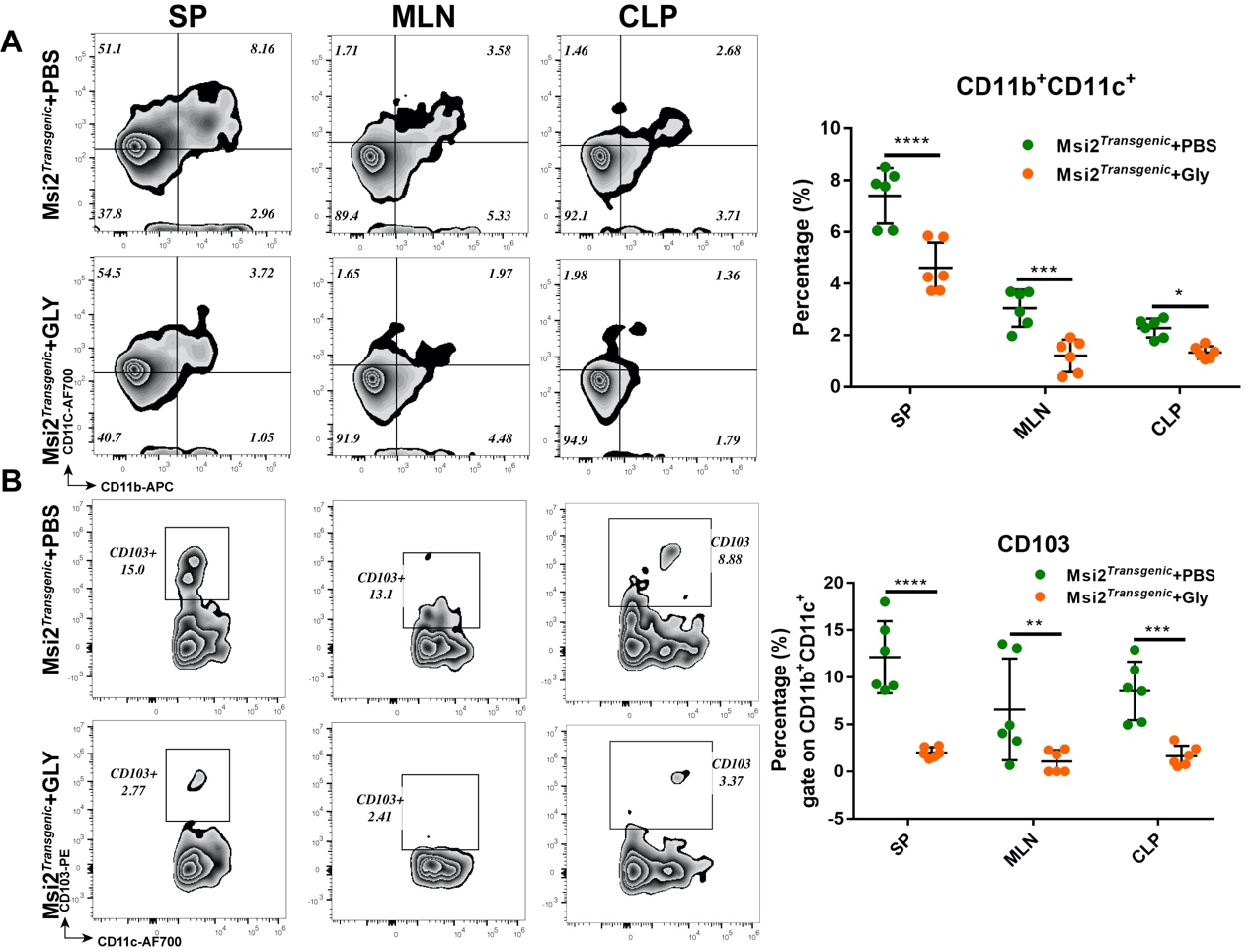


**Fig.S9**

**A**, FACS and statistical analysis of the CD11b^+^CD11c^+^ percentages isolated from Gly treated CAC mice Spleen, MLN and CLP, n=6. **B**, FACS and statistical analysis of the CD11b^+^CD11c^+^CD103^+^ percentages isolated from Gly treated CAC mice Spleen, MLN and CLP, n=6. These results are presented as the mean ± SD values; ns, no significant, **p* < 0.05, ***p* < 0.01, ****p* < 0.001, *****p* < 0.0001; (**A-B**) unpaired 2-tailed Student’s t-test.


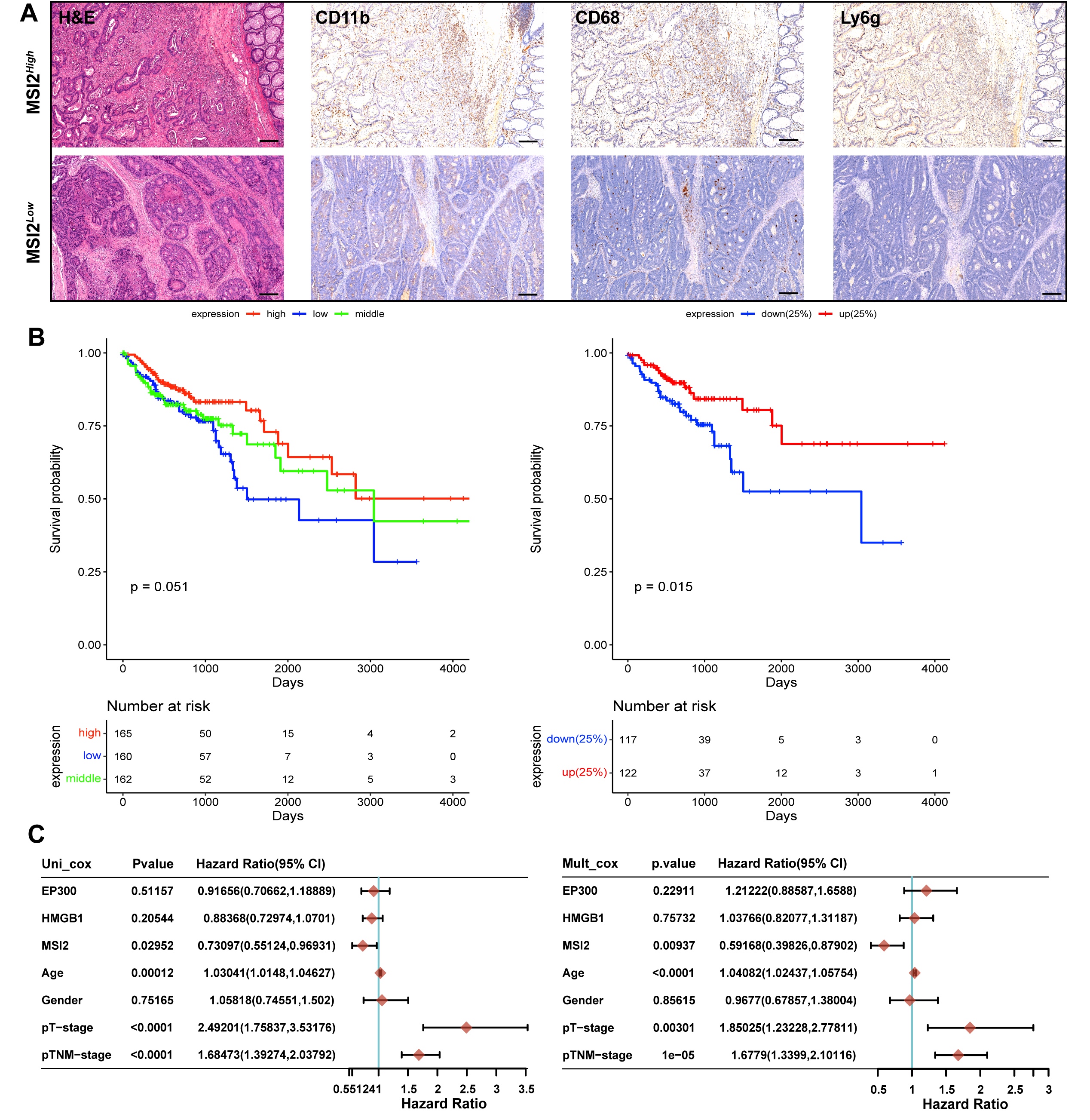


**Fig.S10**

**A**, Representative H&E image and IHC images of CD11b, CD68 and Ly6g in MSI2-high and MSI2-low CRC patient tissues. Scale bars, 200μm. **B**, Kaplan-Meier analysis were performed on the overall survival of three and four groups of CRC patients based on expression of MSI2-FPKM-UQ from GDC TCGA Colon Cancer (COAD). **C**, MSI2, HMGB1 and EP300 expression, clinical information, risk coefficient-Hazards Ratio (HR) and confidence interval were analyzed by univariate and multivariate Cox regression to evaluate 5-year overall survival of CRC patients.

**Supplementary Table S1: Coding genes Primers.**

| **Gene** | **Forward Primer** | **Reverse Primer** |
| --- | --- | --- |
| **Human-β-Actin** | AAGGTGACAGCAGTCGGTT | TGTGTGGACTTGGGAGAGG |
| **Human-MSI2** | ATCCCACTACGAAACGCTCC | GGGGTCAATCGTCTTGGAATC |
| **Human-HMGB1** | TCCTTCGGCCTTCTTCCTCTTCTG | TCGCAACATCACCAATGGACAGG |
| **Human-IL6**  **Human-TNFA**  **Human-IL1B**  **Human-HDAC1**  **Human-HDAC2**  **Human-SIRT1**  **Human-EP300**  **Mouse-Msi2**  **Mouse-Hmgb1**  **Mouse-Il6**  **Mouse-Il1b**  **Mouse-Tnfa**  **Mouse-Ifng**  **Mouse-Il17**  **Mouse-Gapdh**  **Mouse-Il10**  **Mouse-Il33** | ACTCACCTCTTCAGAACGAATTG  CCTCTCTCTAATCAGCCCTCTG  GTCGGAGATTCGTAGCTGGA  CCGCATGACTCATAATTTGCTG  ATGGCGTACAGTCAAGGAGG  TAGCCTTGTCAGATAAGGAAGGA  GCTTCAGACAAGTCTTGGCAT  GCGATGCTGATGTTCGACAA  GGCGAGCATCCTGGCTTATC  TACCACTTCACAAGTCGGAGGC  GCAACTGTTCCTGAACTCAACT  GGTGCCTATGTCTCAGCCTCTT  TCCTCGCCAGACTCGTTTTC  CACCCCCGGAACACCAAAG  AGGTCGGTGTGAACGGATTTG  CCCATTCCTCGTCACGATCTC  TCCAACTCCAAGATTTCCCCG | CCATCTTTGGAAGGTTCAGGTTG  GAGGACCTGGGAGTAGATGAG  ATGATGGCTTATTACAGTGGCAA  ATTGGCTTTGTGAGGGCGATA  TGCGGATTCTATGAGGCTTCA  ACAGCTTCACAGTCAACTTTGT  ACTACCAGATCGCAGCAATTC  TCTCCACAACGTCTTCATTCTCA  GGCTGCTTGTCATCTGCTG  CTGCAAGTGCATCATCGTTGTTC  ATCTTTTGGGGTCCGTCAACT  GCCATAGAACTGATGAGAGGGAG  GTCTTGGGTCATTGCTGGAAG  CATACTCTTCCATTCGAGCGTAG  TGTAGACCATGTAGTTGAGGTCA  TCAGACTGGTTTGGGATAGGTTT  CATGCAGTAGACATGGCAGAA |

**Supplementary Table S2. Antibodies used in Western blot and IFC.**

| **Antibodies** | **Dilution** | **Clone(source)** | **Company (Cat No)** |
| --- | --- | --- | --- |
| **MSI2**  **MSI2** | 1:1000  1:1000/1:200 | Rabbit monoclonal  Mouse monoclonal | Abcam (ab76148)  Abcam (ab156770) |
| **HMGB1**  **HMGB1-Chip grade** | 1:1000/1:200  1:1000/1:200 | Rabbit monoclonal  Rabbit polyclonal | Abcam (ab228624)  Abcam (ab18256) |
| **P65** | 1:1000 | Rabbit polyclonal | Abcam (ab31481) |
| **P-P65** | 1:1000 | Rabbit monoclonal | CST (#3033) |
| **Acetyl-K29-HMGB1** | 1:1000/1:200 | Rabbit polyclonal | Abclone(#A16002) |
| **Acety-lysine**  **P300**  **P105**  **CD11C**  **Laminb1** | 1:1000  1:1000  1:1000  1:200  1:1000 | Mouse monoclonal  Rabbit polyclonal  Rabbit polyclonal  Rabbit polyclonal  Rabbit monoclonal | PTMLab(#PTM-101)  Affinity(#AF5360)  Affinity(#AF6221)  Servicebio(#GB11059)  Abcam (ab133741) |
| **HISTONE H3.1** | 1:1000 | Mouse monoclonal | Sungene biotech (KM9005T) |
| **Goat anti-Rabbit IgG HRP** | 1:5000 | Goat polyclonal | Sungene biotech (LK2003) |
| **Goat anti-Mouse IgG HRP** | 1:5000 | Goat polyclonal | Sungene biotech (LK2001) |
| **Goat anti Rabbit IgG (AF488)** | 1:200 | Goat polyclonal | Abcam (ab150077) |
| **β-Tubulin** | 1:2000 | Mouse monoclonal | Sungene biotech (KM9003T) |
| **β-Actin** | 1:2000 | Mouse monoclonal | Sungene biotech (KM9001T) |
| **Goat anti Mouse IgG (AF594)**  **Flag-tag**  **Myc-tag**  **Myc-HRP**  **Ms anti-Rb light chain HRP**  **Rb anti-Ms light chain HRP**  **Goat anti-Rb heavy chain HRP** | 1:200  1:1000  1:1000  1:5000  1:5000  1:5000  1:5000 | Goat polyclonal  Mouse monoclonal  Mouse monoclonal  Rabbit polyclonal  Mouse monoclonal  Rabbit polyclonal  Goat polyclonal | Abcam (ab150116)  Bioss (bsm-33346M)  Abbkine(A02060)  Abcam (ab1326)  Proteintech (SA00001-7L)  Bioss (bs-0330R)  Abbkine(A25222) |

**Supplementary Table S3. Antibodies used in FACS.**

| **Antibodies** | **Dilution** | **Clone(source)** | **Company (Cat No)** |
| --- | --- | --- | --- |
| **CD45-PE anti-Human**  **CD11c-APC anti-Human**  **CD11b-FITC anti-Human**  **CD80-PE anti-Human**  **CD86-APC anti-Human**  **CD83-FITC anti-Human**  **CD40-AF700 anti-Human**  **CD3-FITC anti-Mouse**  **CD4-FITC anti-Mouse**  **CD4-Percp anti-Mouse**  **CD8a-BV510 anti-Mouse**  **CD103-PE anti-Mouse**  **CD11b-APC anti-Mouse**  **CD11c-AF700 anti-Mouse** | 1:200  1:200  1:200  1:200  1:200  1:200  1:200  1:200  1:200  1:200  1:200  1:200  1:200  1:200 | Mouse  Mouse  Mouse  Mouse  Mouse  Mouse  Mouse  Rat  Rat  Rat  Rat  Mouse  Rat  Ar hamster | BD Pharmingen (555483)  Elabscience (E-AB-F118E)  BD Pharmingen (562793)  BD Pharmingen (560925)  BD Pharmingen (560956)  BD Pharmingen (560929)  BD Pharmingen (561208)  BD Pharmingen (561798)  BD Pharmingen (553046)  Biolegend (303910)  BD Pharmingen (563068)  BD Pharmingen (557495)  BD Pharmingen (553312)  BD Pharmingen (560583) |
| **MHCII-PE-cy7 anti-Mouse** | 1:200 | Rat | eBioscience (25-5321-82) |
| **Fixable viability dye-EF780** | 1:1000 | N/A | Invitrogen (65-0865-14) |

**Supplementary Table S4. WT and Mutation 3^’^UTR of HMGB1 and Primers.**

| **Wild-type**  **HMGB1** | tgaaatttttcttcttgaggggaagctagtcttttgcttttgcccattttgaatcacatgaattattacagtgtttatcctttcatatagttagctaataaaaagcttttgtctacacaccctgcatatcataatgggggtaaagttaagttgagatagttttcatccataactgaacatccaaaatcttgatcagttaa |
| --- | --- |
| **Mutation**  **HMGB1** | tgaaatttttcttcttgaggggaagctagtcttttgcttttgcccattttgaatcacatgaattattacagtgtttatcctttcataaaaaaaactaataaaaagcttttgtctacacaccctgcatatcataatgggggtaaagttaagttgagatagttttcatccataactgaacatccaaaatcttgatcagttaa |
| **T7-HMGB1** | CTAATACGACTCACTATAGGtgaaatttttcttcttgaggggaagctagtcttttgcttttgcccattttgaatcacatgaattattacagtgtttatcctttcatatagttagctaataaaaagcttttgtctacacaccctgcatatcataatgggggtaaagttaagttgagatagttttcatccataactgaacatccaaaatcttgatcagttaa |
| **RIP-Primer-F** | CTTGAGGGGAAGCTAGTCTTTTGC |
| **RIP-Primer-R** | CCCCCATTATGATATGCAGGGTGTG |
